# A Critical Review on Crystal Growth Techniques for Scalable Deposition of Photovoltaic Perovskite Thin Films

**DOI:** 10.3390/ma13214851

**Published:** 2020-10-29

**Authors:** Mazhar Abbas, Linxiang Zeng, Fei Guo, Muhammad Rauf, Xiao-Cong Yuan, Boyuan Cai

**Affiliations:** 1 Nanophotonics Research Center, Shenzhen Key Laboratory of Micro-scale Optical Information Technology, Shenzhen University, Shenzhen 518060, China; mazharszu@outlook.com (M.A.); xcyuan@szu.edu.cn (X.-C.Y.); 2 College of Chemistry and Chemical Engineering, Shanxi Datong University, Datong 037009, China; 11726029@zju.edu.cn; 3 Institute of New Energy Technology, College of Information Science and Technology, Jinan University, Guangzhou 510632, China; fei.guo@jnu.edu.cn; 4 College of Chemistry and Environmental Engineering, Shenzhen University, Shenzhen 518060, China; m.rauf@szu.edu.cn

**Keywords:** perovskite thin films, crystal growth, additive engineering, scalable deposition

## Abstract

Although the efficiency of small-size perovskite solar cells (PSCs) has reached an incredible level of 25.25%, there is still a substantial loss in performance when switching from small size devices to large-scale solar modules. The large efficiency deficit is primarily associated with the big challenge of coating homogeneous, large-area, high-quality thin films via scalable processes. Here, we provide a comprehensive understanding of the nucleation and crystal growth kinetics, which are the key steps for perovskite film formation. Several thin-film crystallization techniques, including antisolvent, hot-casting, vacuum quenching, and gas blowing, are then summarized to distinguish their applications for scalable fabrication of perovskite thin films. In viewing the essential importance of the film morphology on device performance, several strategies including additive engineering, Lewis acid-based approach, solvent annealing, etc., which are capable of modulating the crystal morphology of perovskite film, are discussed. Finally, we summarize the recent progress in the scalable deposition of large-scale perovskite thin film for high-performance devices.

## 1. Introduction

Organic-inorganic hybrid perovskite thin films are extensively investigated in photovoltaic and other optoelectronic devices, including photodetectors, light-emitting diodes, and memory devices, since the last decade [1,2,3,4,5]. This is mainly related to the remarkable intrinsic properties of perovskite semiconductors, such as low exciton binding energy, high absorption coefficient, long charge diffusion length, high charge carrier mobility, abundant cheap material as well as easy and scalable solution manufacturing [2,6,7,8,9]. This unique blend of features enables the perspective of high-efficiency thin-film photovoltaic devices manufactured by solution spin-coating or other low-cost fabrication methods.

At the early stage of hybrid halide perovskite thin film research, the photovoltaic efficiency was found to be very low. The first research development on perovskite solar cells (PSCs) was reported by Kojima et al. using Pb halide perovskite as a sensitizer in photoelectrochemical devices with 3.8% power conversion efficiency (PCE) in 2009 [10]. The major breakthrough in PSCs was the development of solid-state form devices, which were fabricated by Kim et al. and Lee et al. in 2012, with reported PCEs of >9% and >10.9%, respectively [11,12]. In recent years, the intensive and collective research efforts of the photovoltaic community in the direction to improve perovskite thin-film quality and stability [13,14,15,16] has propelled the performance of PSCs, approaching the single-junction silicon solar cells and the fully industrialized indium gallium diselenide (CIGS) solar cell, displaying bright prospects for its commercialization [17,18].

The perovskite thin film is the core component of PSCs, whose quality and stability would directly determine the cell performance [19]. Many reports confirm that fast nucleation followed by slow crystallization of wet film improves the perovskite thin film morphology [20]. A rapid removal of solvent from perovskite precursors increases the supersaturation, leading to the fast nucleation. The antisolvent extraction technique for quick removal of the solvent is the most successful one to improve thin film morphology for lab-scale devices [21]. Along with antisolvent extraction, other physical techniques, such as hot casting, vacuum quenching, and gas blowing, are also used for the rapid removal of solvents and are more compatible in large-area deposition technologies [22]. In addition to the above physical techniques, the morphology of perovskite thin films can effectively be improved by the use of chemical additives in the precursor solution, which slow down the crystallization process, leading to obtaining preferred grain growth and better morphology [23]. Due to the soft ionic nature of perovskites, surface and grain boundary defects are easily produced during thermal annealing. Therefore, chemical additives improve both morphology and stability of PSCs by the defect passivation [24,25]. Similarly, solvent engineering (to influence the solute particle’s nucleation) and perovskite chemical composition are also beneficial for the improvement in device stability and thin film morphology [26,27].

However, by using spin coating and the above crystallization techniques, all record-efficiency PSCs are fabricated on small active areas, nearly 0.1 cm^2^ substrates, which contradicts the desires for PSCs commercialization [20]. For commercialization, it is required to fabricate such high-efficiency solar modules with an active area larger than 800 cm^2^ [28]. It has been reported that the perovskite materials activation energy for nucleation and crystallization (56.6–97.3 kJ·mol^−1^) is sufficiently lower than the amorphous silicon (280–470 kJ·mol^−1^) [29,30]. The low crystallization energy required for perovskite growth enables the deposition of perovskite layers by various low-temperature large-scale fabrication technologies. In recent years, the scalable deposition technologies, including slot-die coating, blade-coating, spray-coating, and ink-jet printing, have been applied for large-area solar module fabrication [31,32,33,34]. Some of the spin coating crystallization techniques are still applicable in large-scale deposition methods; therefore, many research institutions and solar energy companies worldwide have accelerated the pace of the commercialization process of PSCs.

For industrial ambitions, in the past few years many research efforts have been made to scale up the perovskite solar cell devices. A solar module with an active area of over 500 cm^2^ and a PCE of around 12% was fabricated by Solaronix in 2016 [35]. Similarly, another photovoltaic (PV) company, Micro-quanta Semiconductor [36], fabricated a small module with an active area of 17.3 cm^2^ and reported a world record efficiency of 17.9%. Despite the significant advancements in large-scale fabrication technologies, the efficiencies of large-scale perovskite solar cells are substantially lower than the small area devices. The considerable loss in PCE of large-scale PSCs might be attributed to the challenges of uniformly coating large-scale high-quality perovskite films. From this point of view, the in-depth understanding of the overall features for crystallization and redeveloping novel robust deposition technologies that are compatible with large-area uniform and compact thin-film formation is imperative [37].

In this article, we reviewed in detail the fundamental classical nucleation and crystal growth theories and their application in the developed robust crystallization strategies that have been proved to be capable of producing high-quality of perovskite thin film. Furthermore, some of the most promising scalable thin film deposition techniques used for photovoltaic applications are reviewed and discussed.

## 2. **Fundamental Nucleation Theories and Crystal Growth Models**

Homogeneous growth of perovskite thin films is imperative to deliver high efficiency, large and small area PSCs and modules, respectively. The perovskite film growth mechanism is composed of nucleation and crystal growth processes, which play a vital role in the film morphology and the performance of the perovskite PSCs. The fundamental understanding of the factors which influences the nucleation to control the growth of grains in the perovskite crystals is crucial to realize its potential applications. Therefore, in this section, fundamental crystal growth theories and classical nucleation theory are discussed in detail.

### 2.1. LaMer’s Mechanism

**Figure 1:**
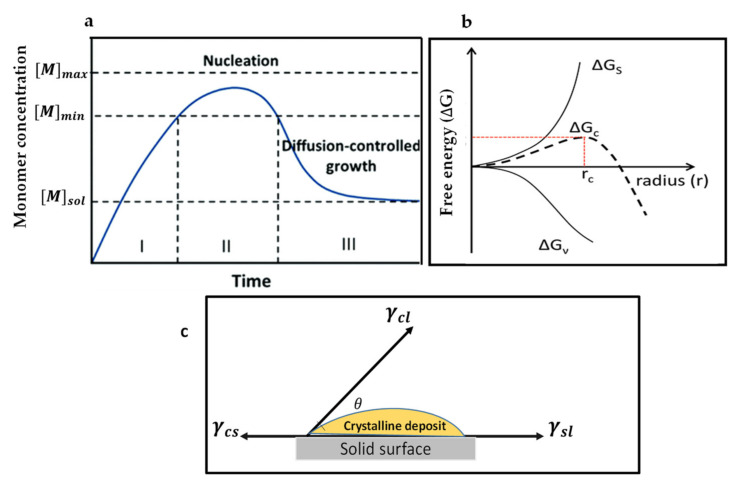
(**a**) LaMer’s diagram illustrates the nucleation and growth kinetics. Reproduced with the permission from ref. [20], Copyright 2020, Chemical Society Reviews. (**b**) Illustration of the nucleation theory. ΔG represents the free energy change and is as the function of nuclear radius (r). Reproduced with the permission from ref. [39], Copyright 2019, Advanced Functional Materials. (**c**) Explanation of the contact angle (*θ*) for heterogeneous nucleation.

The first crystal growth mechanism is LaMer’s mechanism [38,39], which conceptually describes thin film growth in two ways. (a) When the concentration of growing particles falls below the lowest critical concentration required for nucleation, the development of crystals continues, but the nucleation ceases, this is called diffusion-controlled growth. (b) Surface-process-controlled growth: the growth rate can be controlled by the surface process when the diffusion of the growth species from the bulk to the growth surface is adequately fast. LaMer compared the degree of supersaturation of the solution with the nucleation and growth of crystals, which are regarded as the crucial factors deciding the process for film-forming and, subsequently, the film morphology. During annealing, instantly after the deposition of films, the precursor solution concentration increases to approach supersaturation concentration (Cs), where the development of perovskite nuclei begins to occur (stage I). After stage I, the formed nuclei grow with the supply of monomers via diffusion, whereas the nucleation continues to occur (stage II). When the solute is consuming faster than the evaporation rate of the solvent, at this stage, the solution concentration drops below the Cs, and only the growth of the nuclei occurs without the formation of the additional nuclei (stage III). Figure 1a represents the nucleation and the crystal growth process occurring in three stages as per the LaMer mechanism. The crystal growth mechanism of perovskite films can be regulated by controlling either the nucleation or growth.

### 2.2. Von Weimarn’s Theory

Von Weimarn’s theory explains the correlation between the initial supersaturation and the average size of aggregates of a freshly settled phase, which is formed during the nucleation processes in growing systems [40]. Its empirical rules have been applied to comprehend the crystal growth mechanism and have gained fundamental importance in crystal growth theories [41,42]. In brief, the first rule of von Weimarn’s theory argues that, if the initial relative supersaturation decreases, the average crystal size is determined after the crystallization process increases. The second rule suggests that the average crystal size shows a declining tendency as the function of the initial relative supersaturation within a given crystallization time. The first rule is more applicable and well-suited with modern theories as compared to the second rule [41]. On the basis of these rules, nuclei formation and crystal growth are intensely reliant on the supersaturation degree of the solution system. In short, the competition between crystal nucleation and growth can control the average sizes of the crystal clusters. Von Weimarn’s rules can define the rate of nucleation given by (Equation (1) [41]:

(1) $$V_{1}=\mathrm{kAexp}\left(-\frac{\Delta G}{K_{B}T}\right)$$

where A represents the complex function for molecular-level diffusion, T represents the absolute temperature, k_B_ is the Boltzmann’s constant, G and is the critical free energy of the nucleation is represented by ΔG. It can be defined for a spherical perovskite cluster [43], as given by Equation (2):

(2) $$\Delta G=\frac{16{\pi \gamma }^{2}\Omega^{3}}{3K_{B}^{2}T^{2}\delta^{2}}$$

where Ω represents the perovskite molecular volume of the crystal, γ represents the surface free energy of the critical emerged phase, δ reprents the level of the supersaturation of precursors in perovskite solution. According to Equations (1) and (2), V_1_ shows an exponential increasing rate with the level of supersaturation.

### 2.3. Ostwald Ripening

Ostwald ripening [44] is another model to elucidate the particle growth mechanism in a precursor solution, which can be defined as the dissolution of small-sized particles and the redepositing on the surface of larger particles [45,46]. The driving force for this growth process is based on the thermodynamic principle, i.e., the Gibbs-Thomson’s relationship, particles which are smaller in size dissolve quickly because of their larger chemical potential and solubility [47]. A concentration gradient can then be established between particles with different sizes, allowing the existing larger particles to continue growing. Consequently, the concentrations of the growth species can be typically decreased during the Ostwald ripening process, while for the growing particles, the average size increases. The grain growth process in perovskite thin films can be explained by the Ostwald ripening mechanism via coalescence [48,49]. For solution-processed perovskite, the residual solvent molecules, which are embedded in perovskite intermediate films, have a crucial supportive role in the perovskite grain dissolution-recrystallization process during thermal annealing and substantially increase the Ostwald ripening effects. It was stated that perovskite precursor films would become perovskites after releasing the remaining solvent molecules. In the meantime, the small-size perovskite grains can be dissolved in the solvent, and then recrystallize onto the bigger neighboring grains, leading to perovskite grain coarsening by Ostwald.

### 2.4. Fick’s Law of Diffusion

Fick’s law describes the growth of particles on the growing site from the solution by diffusion process (motion of particles from a region of higher to lower concentration), which comprises two methods: (1) transport of particles from the solution to the crystal surface, (2) the reaction of the growing species on the surface. In the first step, according to Fick’s first law [50], the particles diffusion flux is stated as:

(3) $$J=4{\pi r}^{2}D\frac{d\left[M\right]}{\mathrm{dx}}$$

Here, J represents the total flux of particles, r is the radius of particles, D is the diffusion coefficient, and [M] is for the concentration of particles. The concentration gradient around a spherical particle surface can be further derived from the total flux of monomers (J) according to the diffusion-controlled growth.

(4) $$J=4{\pi r}^{2}K({\left[M\right]}_{s}-\left[M{]}_{r}\right)$$

Here, the rate of surface reaction is denoted by k, the solute concentration at the particle/solution interface is represented by [M]_s_, and [M]_r_ stands for the solubility of particle. Larger particles are less soluble and grow more quickly than smaller ones as per the Gibbs–Thomson effect. Based on Equations (3) and (4), it can be concluded that the particle growth obviously depends on either the surface reaction or the diffusion process. Thanks to the steady-state of the solute diffusion, the controlled crystal growth can enhance the realization of the homogeneous particles, despite the fact that the monomer concentration at the surface of a particle can reach a value equivalent to that of a bulk solution due to the slow surface reaction (as shown in Figure 1a). Recently, Liu et al. [51] judiciously explored the cation diffusion method following Fick’s second law before the perovskite crystallization at the gel stage by implementing the diffusion model responsible for the phase segregation. They demonstrated the uniform 2D perovskite with the formula of (BA_2_MA_3_Pb_4_I_13_) thin-film without noteworthy phase segregation. Their results reveal that the local phase distribution in perovskite materials, regardless of the 2D or 3D composition, was very reliant on the gel stage kinetically controlled via diffusion.

### 2.5. Classical Nucleation and Perovskite Crystal Growth

According to the classical nucleation theory, a nucleus can be regarded as a sphere of the condensed phase [52], and nucleation of particles can be defined as the process in which nuclei with a definite thermodynamic phase act as templates for the crystal to grow [53]. Nucleation takes place when the growing species concentration is adequately higher than the solubility to attain a supersaturated state. Consequently, supersaturation has a significant function in stimulating the process of nucleation. To understand the process of nucleation, the shape of the particle was described as a sphere like a ball. In this case for a spherical particle, the total free energy, which represents the free energy required to stabilize nuclei without dissolving in the solution, could be used to describe the nucleation rate. It is the sum of the bulk-free energy ($\Delta G_{V}$) (which is the free energy between large-size particles and the solute in solution) and the surface free energy ($\Delta G_{S}$) (which is between the surface particle and the bulk particle). Furthermore, $\Delta G_{S}$ is a positive value proportional to r^2^ (particle radius) and $\Delta G_{V}$ is a negative value proportional to r^3^ when approaching the supersaturated state. Understandably, the formation of nuclei in the phase of the supersaturated solution depends mainly on the nuclei critical radius (r_c_) [47]. The nuclei with a radius smaller than the critical radius (r_c_) have to redissolve into the solution; however, if the radius of nuclei is larger than r_c_, they can be thermodynamically stable and may exist in the solution. Critical radius (r_c_) can, therefore, be regarded as the minimum radius in which a nucleus can persist in the solution and continue to grow. For homogeneous nucleation, critical total free energy is given as:

(5) $$\Delta G_{\left(c\right)}=\Delta G_{s}+\Delta G_{v}=4{\pi r}^{2}\gamma +\frac{4}{3}{\pi r}^{3}\Delta G_{V}$$

Here, γ represents the surface free energy per unit area, which is surface energy between the supersaturated solution and the crystal surface [54]. The bulk-free energy (ΔGv) per unit volume also depends on the temperature (T), the Boltzmann constant (k_B_, degree of supersaturation (S), and molar volume of the nucleus (v). The degree of supersaturation can be written as S = C/C_S,_ where C represents the solute concentration, while Cs represents the solubility limit in the case of supersaturation (C > Cs). Therefore, ΔGv can be written as:

(6) $$\Delta G_{V}=-\frac{K_{B}\mathrm{Tln}\;S}{V_{m}}$$

Here, V_m_ stands for the monomer’s molar volume in the crystal, considering the surface free energy (4πr^2^γ, positive) and bulk-free energy (4/3 πr^3^ΔG_V_, negative), ΔG(c) is given in Figure 1b. When the nuclei are smaller than r_c_, they can only redissolve into solution. Therefore, the critical radius can be the minimum size of the nuclei. For homogeneous nucleation, the nucleation rate can be denoted by the Arrhenius type equation [47]:

(7) $$\frac{\mathrm{dN}}{\mathrm{dt}}=\mathrm{Aexp}\left[\frac{-\Delta G_{N}}{K_{B}T}\right]=\mathrm{Aexp}\left[-\frac{16{\pi \gamma }^{3}V^{2}}{3K_{B}^{3}T^{3}N_{A}^{2}{\left(\mathrm{lnS}\right)}^{2}}\right]$$

Here, N represents the number of nuclei, V^2^ is the molar volume of nuclei, A represents a pre-exponential factor, k_B_ is called as the Boltzmann’s constant, N_A_ represents the Avogadro’s number, S represents the degree of supersaturation, and T represents the temperature. From Equation (3), it can be concluded that the rate of nucleation is strongly influenced by the level of supersaturation [47], surface free energy, and temperature. Moreover, the nucleation rate can be tuned by optimizing the above factors (i.e., high supersaturation, high temperature, and lower surface-free energy). Most of the recently developed methods to improve the quality of perovskite films will be systematically analyzed according to these fundamentals. However, in an actual study, there could be the possibility of the presence of impurities in precursor solutions—the impurity centers which may come from other phases in precursor can reduce the energy barrier, thereby facilitating the occurrence of nucleation [55,56]. Therefore, the heterogeneous nucleation generally occurs considerably easily at such favored sites as compared with the homogeneous nucleation. As the value of ΔGs is always positive and that of ΔGv is negative, the maximum free energy essentially is required to form a stable nucleus, which leads to promoting more growth. When the maximum value of free energy is reached, dΔG/dr should be zero, yielding the critical free energy (ΔG_c_). The interfacial energy diagram has three phases among a liquid and two solids in contacts, as shown in Figure 1c. The interfacial energies between the liquid and crystalline phases, crystalline phase and solid surface, solid surface, and liquid are represented by the terms γcl, γcs, and γsl, respectively. The contact angle on the solid surface is represented by θ. If θ < 180°, then there is a high-affinity between nuclei and active centers, which is beneficial to lower the energy barrier to the occurrence of nucleation. The process can be attributed to the substantial drop in the interface energy. Consequently, the free energy for heterogeneous nucleation can be adjusted via using the term ∅, a factor reliant upon θ. This alteration is similar to that of a homogeneous nucleation process, as defined in Equation (8):

(8) $$\Delta G_{c}^{\mathrm{hetro}}=\emptyset \Delta G_{c}^{\mathrm{homo}}$$

(9) $$\emptyset =\frac{(2+\cos\theta ){\left(1-\cos\theta \right)}^{2}}{4}$$

When the contact angle of the solution *θ* = 180° (involving no affinity among the solid surface and solution at all), then cos *θ* = −1 and φ = 1, hence, $\Delta G_{c}^{hetro}=\Delta G_{c}^{homo}$, which implies that both the heterogeneous and homogeneous nucleation critical energies are equivalent. Equation (8) explains that the contact angle of the perovskite precursor on the substrate (heterogeneous nucleation site) can affect the rate of nucleation. Furthermore, when the precursor solution is prepared by using polar solvents, then the hydrophobic substrates ∅ becomes larger, which slows down the rate of nucleation. Cheng et al. [57] revealed that a larger contact angle of perovskite solution on the hydrophobic surface could effectively suppress perovskite film’s nucleation and facilitate the development of a large-size grain because of the improved grain boundary mobility. In their study, they used the hydrophobic substrate for the growth of a thin single-crystal perovskite film. Both the improved ion diffusion on the hydrophobic surface and suppressed nucleation assisted the growth of large-area films [58].

Equations (7) and (9) illustrate that there are four factors that can be managed experimentally to alter the activation energy of the nucleation, which are wettability of the substrate, surface energy, supersaturation level, and temperature. In the perovskite film formation process, the traditional spin coating technique normally produces an inadequate supersaturation because of the moderate rate of evaporation of the most frequently employed solvents for perovskite precursors such as dimethyl sulfoxide (DMSO), (dimethylformamide (DMF), and dimethylacetamide (DMAc), which leads to a low density of heterogeneous nuclei. Alternatively, the crystal growth rate is comparatively fast depending on the solution supersaturation, promoting rapid solute precipitation at those regions with high surface energy. The difference between nucleation and growth rate speeds up the development of large dendritic perovskite structures on very few numbers of nuclei, which can be harmful to perovskite device efficiency. To make a compact perovskite layer with full surface coverage, it is imperative to achieve a high nucleation rate before the crystal growth starts. The most reliable and successful method to improve the nucleation-growth is by influencing the nucleation, using various techniques such as the use of antisolvents, composition engineering, additive engineering, and physical methods. The purpose of both these approaches is to decelerate the crystal growth process and assist nucleation. These approaches are outlined in the following section.

## 3. Crystallization Techniques for Depositing High-Quality Perovskite Thin Films and Photovoltaic Devices

Based on the analysis of crystal growth models and classical nucleation theory, the nucleation and crystal growth process can influence perovskite films’ morphology. In general, rapid nucleation, followed by the slow growth of the crystal, is necessary for the development of uniform, pin-hole, and defect-free high-quality perovskite films. In the last few years, various advanced methods have been reported to make perovskite films’ quality better as per the above factors, which are presented in this section.

### 3.1. Antisolvents for Fast Nucleation

Nonpolar solvents that are miscible with host solvents such as DMF and DMSO, but do not dissolve the perovskite precursors, are utilized in perovskite solution. In the one-step coating, the control on the rate of nucleation is relatively challenging as the reaction takes place in a relatively short time due to the fast removal of solvents. Consequently, the device’s reproducibility is identified to be low compared with the devices than the two-step process [59]. The antisolvents can be applied in the spin coating to improve the morphology of perovskite films, which causes the solvents to be extracted quickly from the precursor solutions and gives rise to the fast supersaturation of solute in the perovskite precursor film. Antisolvent dripping is used extensively in the small lab-scale best-performing PSCs [60].

**Figure 2:**
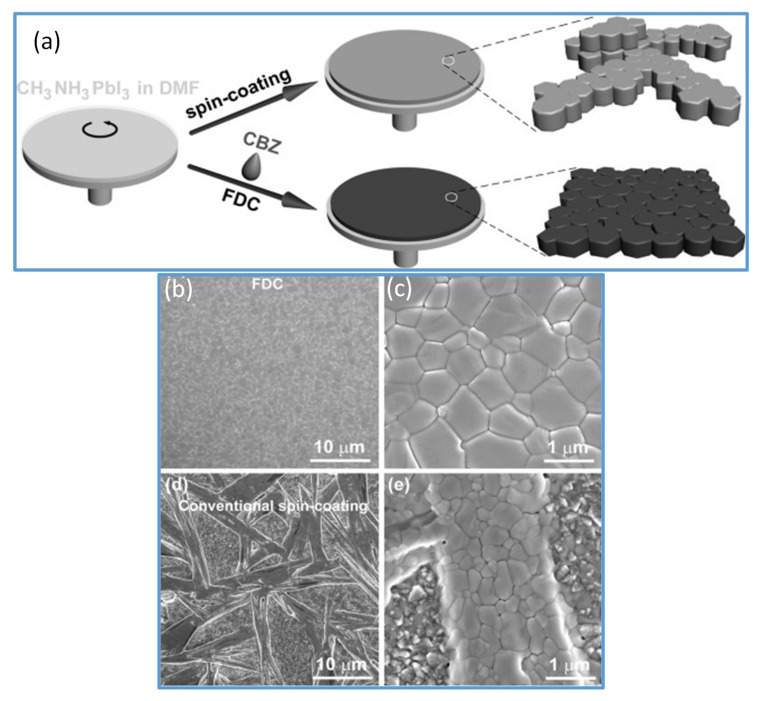
(**a**) Represents the spin-coating process and the FDC process to fabricate perovskite films. Spin coating (top) produces a shiny gray film with nonuniform large crystals. A second solvent (CB) is dripped on the wet film during the spin coating to induce fast crystallization producing the uniform size perovskite grains. (**b**,**c**) SEM images of CH_3_NH_3_PbI_3_ film fabricated by FDC process using antisolvent (CB). (**d**,**e**) SEM images of a film fabricated by conventional spin coating. Reproduced with the permission from ref. [61], Copyright 2014, *Agewandte Intl. Ed. Chemie*.

In 2014, Seok et al. [59] introduced this technique in spin coating the perovskite films; since then, it is generally used to achieve smooth and dense perovskite thin films. The objective of solvent soaking techniques is to create uniform nucleation all over the surface of films. Similarly, Xiao et al. further tested the dropping of chlorobenzene (CB) in the course of a spin coating deposition of perovskite solution, resulting in a fast and uniform nucleation across the film, which yielded a super quality, dense, homogeneous, and completely covered perovskite films [61] (as shown in Figure 2a). It is described that CB quickly removes extra DMF solvent and decreases the solubility of the perovskite in the precursor wet film. This will lead to rapidly created supersaturation of the solute, which further encourages the formation of more nuclei, leading to a homogenous perovskite film (Figure 2b,c) with good surface coverage and reproducibility. SEM images of perovskite films fabricated by conventional spin coating are shown in Figure 2d,e for comparison. Based on the achievements of this approach, diverse, environmentally friendly green antisolvent alternatives such as chloroform (CF), toluene [62], chlorobenzene [61], hexane [63], ethyl acetate [64], anisole [65], ethyl ether [66], and mixed ones have been investigated, in coordination with different perovskite formulations and variations of host solvents [67]. A large number of solvents with different physicochemical properties and dielectric constants are systematically studied to reach a more accurate antisolvent selection protocol. The solvents such as CB, toluene (Tol), p-xylene (Xyl), diethyl ether (DE), trifluorotoluene (TFT), and dichloromethane (DCM) are studied. It has been reported that those antisolvents that are completely soluble with the host solvents, and have high both the dielectric constants and boiling points, tend to form smooth superior quality films with well-balanced nucleation and substrate coverage [60]. In recent years, Wang et al. [68] demonstrated the mechanism of green solvents to realize an intermediate phase-controlled FA-based high-efficiency PSCs. They introduced several antisolvents including anisole, diethyl ether (DE), dibutyl ether (DBE), and diisopropyl ether (DIE), in the one-step fabrication of perovskite films from the FA-based perovskite solution. In their analysis, they proposed that during the precipitation crystallization, the intermediate phase is formed and could be identified and controlled through a judicious selection of the polarity of antisolvents. Knowing the intermediate phase formation mechanism has led them to recognize DIE as a green antisolvent with universal perovskite compatibility. They utilized Cs/FA, FA/MA, and Cs/FA/MA, perovskite precursor to achieve champion PCEs of 20.05%, 20.15%, and 21.26%, respectively, with improved repeatability. Their large-area (1 cm^2^) PSCs showed a PCE of 18.51%.

**Figure 3:**
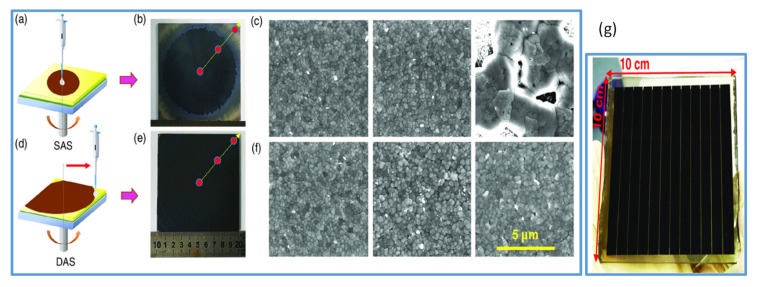
(**a**) Represents the spin coating with static antisolvent (SAS) process. (**b**) The photo of 10 × 10 cm^2^ perovskite film by SAS process. (**c**) SEM images of perovskite film from the left to right by SAS process. (**d**) Spin coating with dynamic antisolvent (DAS) process. (**e**) The photo of the 10 × 10 cm^2^ large perovskite film by DAS process. (**f**) SEM images of large 10 × 10 cm^2^ perovskite films fabricated by DAS process. (**g**) The photo of the solar module by DAS process. Reproduced with permission from ref. [21], Copyright 2019, *Solar RRL*.

Recently, the perovskite research communities have devoted their efforts to improving the efficiency of the large-area PSCs employing various methods to deposit large-area perovskite films. In 2017, Bu and coworkers employed the green solvent ethyl acetate both in perovskite by antisolvent technique and in the hole transport layer (HTL) during its deposition. By this technique, they improved the quality of perovskite films and the HTL layer, which resulted in a high-efficiency PSC with a PCE of 19.4% in small devices and 14.2% for a module of an area 5 × 5 cm^2^ [69]. Similarly, in another work, Tongle Bu et al. [21] reported an antisolvent quenching method called dynamic antisolvent (DAS) in the spin coating process to realize large-area smooth perovskite films to achieve high-efficiency perovskite solar modules (as shown in Figure 3d–f). In this simple method, compact and smooth films from mixed-cation perovskite precursors are fabricated. Moreover, the uniformity of the film across the center to edge is significantly improved as compared to the common solvent engineering (Figure 3b,c,e,f for comparison). Thus, top-performing modules (10 × 10 cm^2^) delivering PCE as high as 17.82% (Figure 3g) and another with a certified efficiency of 17.4% in an aperture area of 53.64 cm^2^ are obtained. The spraying of an antisolvent that is quite similar to the antisolvent dripping is used to produce high-efficiency PSCs by two-step deposition [70].

Besides, the antisolvent spraying process is more suitable for the large-area fabrication of PSCs and, therefore, more beneficial than antisolvent dripping. By spraying antisolvent, a large area (16 cm^2^) PSCs producing efficiency of up to 12.1% is reported [71]. The antisolvent bathing method was developed to extend the antisolvent strategy to scalable deposition techniques; the perovskite precursor film can be soaked in the antisolvent bath for a short period of time [66]. These are the main achievements in large-area PSCs by the application of the antisolvent technique.

### 3.2. Hot Casting

In the perovskite film forming process, thermal annealing is generally needed to crystallize the wet perovskite-coated thin films. Wet film annealing is a physical scheme that can improve the solvent molecules’ evaporation rate in the process of the solution-to-solid phase conversion, which can influence the level of supersaturation and accordingly perovskite nucleation process. The heat treatment is typically done at a moderate temperature of around 100 °C for up to a maximum of 1 h to evade the decay of the perovskite [6,10,72,73,74]. Kim et al., based on this general protocol, stated that the high-temperature annealing for few seconds was promising for the growth of perovskite films. A little high temperature could be beneficial for immediate and quick solvent removal to generate a supersaturation for the solute. The precursor supersaturation speeds up the nucleation formation process and endorses a uniform perovskite deposition for efficient PSCs. Therefore, the grain size can be increased from around 300 nm to more than 1 μm by annealing the as-cast perovskite film under 400 °C just for 4 s [75]. In another similar approach, in 2015, Nie and coworkers reported a hot-casting precursor solution to produce continuous, compact, and large grain size perovskite thin films. Hysteresis-free PSCs with PCE of around 18% were achieved, with excellent reproducibility [76]. This technique can be used both ways; either the substrate is heated to a higher temperature or the precursor solution for the rapid evaporation of the solvents. Compared to other methods, it is relatively simple to heat the substrate or the perovskite precursor ink during deposition, which allows this heating method to be used extensively to adjust the thin film morphology. During hot-blade coating [77], in addition, to speed up the rate of solvent extraction, the high temperature could also encourage the solute atoms to decrease the diffusion energy barrier quickly and thus lead to fast grain formation process to achieve a favorable grain size. Employing this technique, a high efficiency (up to 20.3%) [78] was obtained.

### 3.3. Vacuum Quenching

Many research reports confirm the positive effect of vacuum quenching, which promptly creates supersaturation of the perovskite solute. In 2019, Guo et al. [37] proposed a vacuum-assisted crystallization method for obtaining low-temperature-fabricated perovskite thin films. In this technique, thin films are deposited by blade coating at room temperature instead of elevated temperature. The freshly blade-coated wet films are placed in a vacuum chamber for 2 min then immediately moved for annealing at 100 °C for 10 min to crystallize the films completely. Uniform films with complete substrate coverage and pinhole-free films can be fabricated by this method. Importantly, this technique is quite different than the blade coating at elevated temperatures very close to the boiling point of solvents [79,80,81,82,83]. In another study, Amir et al. [84] proposed the vacuum-controlled meniscus coating, where an optimally performing perovskite film was obtained. They placed the as-coated wet perovskite precursor films in a vacuum chamber for a very short time of only 30 s to keenly remove the solvent in wet film. By this technique, and a natural drying process, they obtained uniform, low surface coverage films with a needle-like morphology. Furthermore, certain analogical approaches, such as gas blowing, solvent annealing, and vacuum flash-assisted solution process (VASP) methods are also reported. A vacuum flash drying method to remove the solvent has been reported many times. Huang et al. [85] used the flow of Ar gas over a spin-coated film to accelerate the removal of solvent. Consequently, they quickly achieved supersaturation, which facilitated the development of further nuclei, converting dendritic shape of crystal with low surface coverage to uniform grains. Therefore, the gas-assisted fabrication of perovskite solar cells demonstrates substantially improved photovoltaic performance as compared to a conventional spin coating process. Using this approach for large-area (>1 cm^2^) PSCs, PCE values > 20% have been achieved [86]. A system has been developed with further improvement to simultaneously conduct low pressure as well as gas blowing on the wet film; the combined effect of vacuum and gas blowing improves the rate of elimination of solvents [87].

### 3.4. Gas Blowing

Gas blowing is another still successful physical approach used to produce uniform perovskite films for PSCs. It is mostly used for controlling the drying kinetics during fabrication of thin films. Usually the nitrogen gas (N_2_) is applied in the gas-blowing process. This technique basically accelerates the rate of evaporation of the precursor solvents and increases the level of supersaturation, thereby regulating the perovskite nucleation and grain growth process. The morphology of the crystallized perovskite thin films is strongly influenced by the rate at which the gas is blowing, therefore, its effect is investigated in many early studies [88,89,90]. By using a gas blowing technique, Li et al. demonstrated antisolvent-free, room temperature, meniscus-coated, uniform, compact, and homogeneous perovskite films under ambient conditions. They reported an air-knife film drying mechanism to fabricate hysteresis-free PSCs with a PCE of 20.26% and 18.76% for an active area of 0.06 cm^2^ and for 1 cm^2^, respectively [91].

In summary, the quick removal of solvent from precursors coated on substrate increases the supersaturation degree, which results in fast nucleation and precipitation of the perovskite materials. The first rapid removal of the solvent was realized by dripping the antisolvent onto a spinning perovskite precursor coated substrate. This technique encourages uniform growth and avoids the formation of large dendritic structures. Nevertheless, the dripping of antisolvents in spin coating generates a complex structure throughout the substrate, and better results were obtained with the spray of the antisolvent. Antisolvent spraying was applied to fabricate large area (16 cm^2^) PSCs and obtained a PCE of 12.1% in its single cell [71]. However, the bottleneck of both antisolvent dripping and spraying is that these techniques are only able to be used in spin coating, and they are not capable of being implemented in a scalable deposition. In further studies, an antisolvent bath approach was proposed, in which the precursor film is soaked in a bath of antisolvent for a short period of time [66]. Although antisolvent bathing is compatible with roll-to-roll continuous fabrication, it is inconvenient to place a substrate with perovskite in a solvent bath for some time. Therefore, further physical techniques (such as hot-casting, gas blowing, vacuum-assisted drying, or a combination of these physical approaches) compatible with scalable deposition were explored. Similarly, smooth, dense, and without pin-hole perovskite thin films were obtained by heating precursor solution or the substrate close to the boiling point of the perovskite precursor solvent in blade coating [77]. However, the elevated temperature will generate point defects because of the volatile nature of the organic component of perovskite. Therefore, vacuum-assisted crystallization is reported in room temperature blade and inkjet printing [37]. Similarly, the gas flow was first utilized to encourage fast crystallization in spin coating. This idea was later employed for slot-die coating via an air blade to assist solvent drying. Another concept to quickly extract the solvent and dry the wet films is the vacuum flash method which utilizes a vacuum for the complete extraction of solvent. The large-area (>1 cm^2^) PSCs with PCEs > 20% are fabricated by using the vacuum-assisted drying technique. In another work, low pressure and strong gas flow were applied simultaneously on a wet substrate. Their combined effect facilitated the increase of a solvent removal rate. Therefore, it is concluded that the combined effect of the above techniques may give better results.

## 4. Strategies to Improve Crystal Morphology of Perovskite Thin Film

The performance of PSCs depends greatly on the morphology of the perovskite thin layers. As mentioned in Section 3 the morphology of perovskite thin films is controlled by fast nucleation of perovskite precursors followed by slow crystallization of wet films. Fast nucleation is induced by quickly removing the host solvent by using antisolvents (followed by gas blowing, vacuum quenching, etc.) during the spin coating method as described in the previous section. Similarly, various techniques have been reported which can efficiently slow down the crystallization process. In this section, we will review strategies (such as the use of the additive, Lewis acid-base adduct formation, solvent annealing, solvent additives, etc.) used to improve perovskite crystal morphology by slowing down the crystallization process.

### 4.1. Use of Additives

The chemistry of the perovskite precursors can greatly influence the nucleation process to facilitate preferred grain growth leading to obtaining a better morphology. For example, adding a small amount of organic or inorganic chemicals in precursor or changing the perovskite’s chemical composition. The high-quality perovskite films are prepared for mini-modules and large area PSCs by applying this technique, so-called additive engineering. It has been widely reported that the use of additives to the perovskite precursor solution could be a useful and consistent method to achieve large size grains in perovskite crystal, suppresses defects, and improves instability, which ultimately enhances the PCE of PSC devices. A large variety of chemical additive has been reported depending on the perovskite composition and the processing route of additives. The additives which have delivered better results include acids (such as HI, HBr, or HCl), metal cations, fullerene derivatives, Lewis acids and bases, surfactants, ammonium salts, and excess amounts of MAI and PbI_2_ in the precursor solution. Marks et al. used a small quantity of liquid additives HBr, HI, and HCl in the perovskite solution to study their effect on phase transition at room temperature. These additives not only passivate surface defects but also change the optical properties of MAPbI_3_ optical properties of perovskite [92,93]. Hydrohalic acids used as additive have a two-fold effect of influencing the perovskite film growth: first, additional anions, available by the addition of hydrohalic acids, coordinate with lead ions, and create an intermediate film after the extraction of solvent and consequently suppresses the nucleation of perovskite; [94] secondly, the PH value of the perovskite precursor solution also decreases by the addition of hydrohalic acids which break down smaller clusters or nuclei, thereby considerably suppressing nucleation and crystal growth of individual domains [95]. Moreover, it is described that perovskite thin films coated from a perovskite precursor solution with little extra chloride ions (MAI + PbI_2_Cl) can exhibit full surface coverage with larger size grains compared to those achieved from a stoichiometric precursor (PbI_2_ + MAI) [12]. The morphology of perovskite films fabricated from the solution having a small number of extra chloride ion provided by additives in perovskite solutions is identical to those obtained with PbCl_2_ [96,97,98]. Ke et al. used lead thiocyanate (Pb(SCN)_2_) as an additive in the perovskite precursor to fabricate large grain perovskite thin films. They assumed that MA^+^ cations and SCN^−^ anions would react during the perovskite film formation and form HSCN and MA gases, which might support the grain growth and the presence of PbI_2_ at grain boundaries passivate the defects at GBs, by decreasing dark current [99]. Additionally, surfactants can also be applied in the small as well as large-area fabrication of perovskite films. Huang et al. reported the addition of a small amount of surfactant (L-α-Phosphatidylcholine) into the perovskite (MAPbI_3_/DMF) precursor solution and blade coated the perovskite films. They demonstrated that the surfactant not only changed the fluid drying dynamics and suppressed the solution flow but also improved the wettability of the perovskite ink on the nonwetting charge transport perovskite films. Furthermore, it also passivated the surface charge defects, and the devices produced over 20% PCE for small-area PSCs and a 15.3% PCE for large area (33.0 cm^2^) perovskite modules [78]. The use of metallic salts as an additive in perovskite precursor solution to improve the quality of perovskite thin films is also reported by several groups [100]. The addition of a small amount of small in FA or MA-based perovskite precursor solution can partially replace FA^+^ or MA^+^ cations, and it can passivate the defects to produce high-quality 3D perovskite films and thereby improve the efficiency and stability. Similarly, other alkali metal cations such as Rb^+^, K^+^, and Na^+^ are also used in perovskite precursors in the form of metal salt. However, these alkali metal ions are only used as an additive to improve perovskite crystal morphology by defect passivation. Their size is much smaller than Cs^+^, and therefore do not meet the requirement to form a 3D perovskite. Chu and colleagues reported KCl, NaCl, and LiCl as additives to fabricate perovskite film by a two-step method, and both KCl and NaCl led to significantly improved PCEs [101].

**Figure 4:**
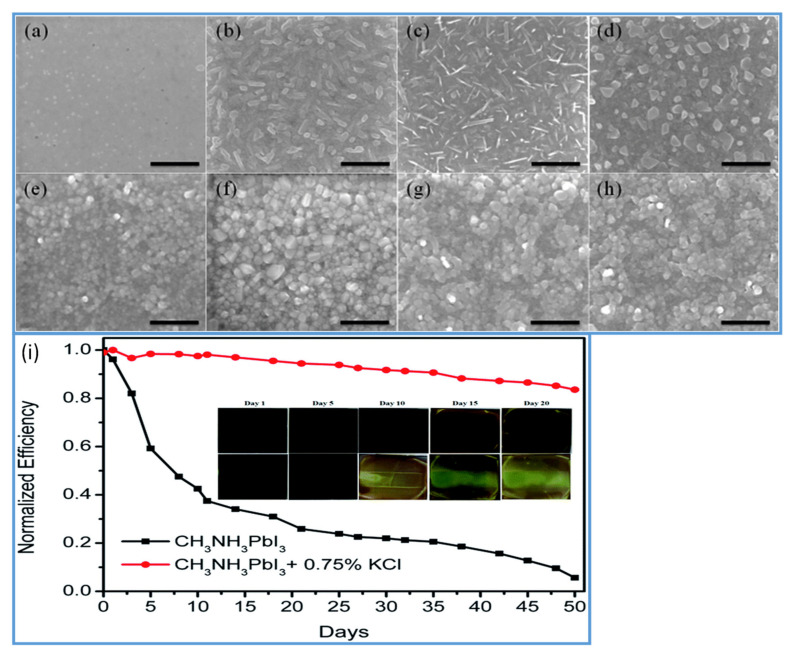
(**a**–**d**) SEM images of PbI_2_ films and (**e**–**g**) SEM of images perovskite films. (**a** and **e**) prepared without additives. (**b**,**f**) with KCl, (**c**,**g**) with NaCl, and (**d**,**h**) with LiCl additives. (**i**) Normalized PCEs as a function of days, of PSCs fabricated with and without KCl as an additive. Inset: color changing images of the perovskite films in air with time. Reproduced with permission from ref. [101], *Journal of Materials Chemistry A*.

In the two-step method, spin-coated PbI_2_ and perovskite films prepared without salt additives are noncontinuous with many small pinholes, as shown in Figure 4a,e. Noncontinuous PbI_2_ films with small crystallite perovskite grains significantly reduce the device performance. More uniform, with large grain size PbI_2_ and perovskite films can be achieved by using 0.75% KCl salt additive (Figure 4b,f) as compared to the films prepared with 1% NaCl (Figure 4c,g) and 0.25% LiCl (Figure 4d,h). The formation of large grains minimizes the permeability of oxygen and moisture and, thereby, enhances the stability of the device (see Figure 4i). Water as an additive is also investigated in PSCs. It has been reported by some research groups that H_2_O has harmful effects on perovskite solar cells, as perovskite films easily decompose under a humid environment [102]. In contrast to this, some groups found that an optimized amount of water could support nucleation and crystallization of the perovskite material; as a result, good quality perovskite films were obtained [103]. Chang et al. stated the synergistic effect of the H_2_O additive and DMF vapor treatment via a two-step spin coating method and fabricated superior quality perovskite films [104]. They demonstrated that a small amount of H_2_O as additives enabled MAI to penetrate the PbI_2_ films to form a thick film with a pure MAPbI_3_ phase and produced large-sized grains by decelerating the crystallization rate of perovskite films. Consequently, they successfully fabricated the high-quality perovskite films and achieved a PCE of 16.7% in a small device of 1.3 cm^2^ and 15.4% PCE for the mini-perovskite modules of area 11.25 cm^2^.

### 4.2. Slow Down Crystallization by Lewis Acid-Base Adduct Formation

The crystallization of perovskites could be slowed down by a simple and practical scheme through a Lewis acid-base adduct formation. An adduct is formed when some base molecule is mixed in the stoichiometric value of perovskite components. Based on the Lewis base theory, an electron pair donating molecules are the bases, while electron-pair accepting molecules are acids [105,106,107,108,109]. Therefore, electron-pair donors such as, oxygen, sulfur, nitrogen-containing monodentate, or bidentate ligands can be used as additives in perovskite precursors, which make an adduct with PbX_2_. Being strong Lewis acids, Lead (II) halides can easily make adducts with Lewis base molecules. The resulting Lewis acid-base adducts in the precursor solution can raise the solubility of lead halides, slowing down the nucleation process and the growth of crystals. Therefore, by controlling the degree of interaction between Lewis acid-base species using specific Lewis base molecules, the rate of nucleation and crystal growth process can be regulated, and finally, the better-quality perovskite thin films can be realized [59,110,111].

**Figure 5:**
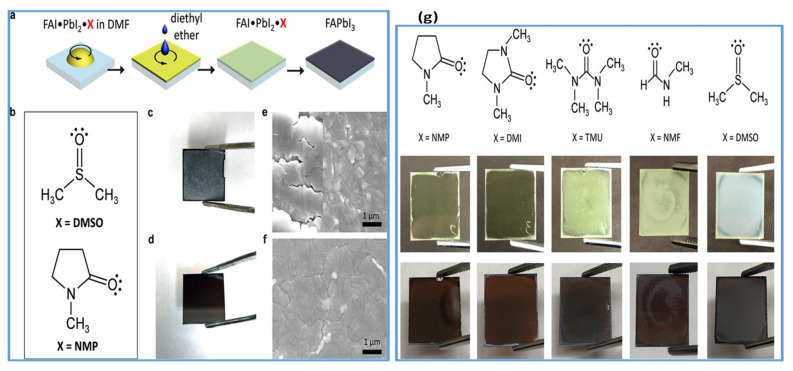
(**a**) Represents the fabrication of FAPbI_3_ perovskite films by a Lewis base adduct approach. (**b**) Represents the molecular structure of Lewis bases: dimethyl sulfoxide (DMSO) and *N*-methyl-2-pyrrolidone (NMP). (**c**–**f**) Photographs and SEM images of theFAPbI_3_ perovskite films. (**g**). Molecular structures of the Lewis bases used in theFAPbI_3_ perovskite films (upper panel) Lewis bases perovskite adducts (middle panel) and the crystallized perovskite films (lower panel). Reproduced with permission from ref. [109], Copyright 2018, *Journal of the American Chemical Society*.

In recent years, DMSO is widely used as an O-donor Lewis base [105,107,109,112,113]. Hence, DMSO can readily form an adduct (PbI_2_–DMSO–MAI) with perovskite due to its strong basicity compared with DMF. During the competition reaction, this intermediate phase will be established earlier than the change of perovskite precursors to solid crystalline perovskite. The existence of an intermediate phase between DMSO and MAPbI_3_ perovskite solution is verified by the Park group using infrared spectroscopy technique [105]. In addition to the antisolvent treatment by diethyl ether, intermediate adduct development essentially helps to make ultra-uniform perovskite films. Perovskite films show rod-shaped morphology with incomplete substrate coverage when fabricated without antisolvent treatment. The lower film morphology is caused by the solubility difference between MAI and PbI_2_ in the host solvent, such as DMF. Therefore, to improve the morphology, another technique was the use of nonpolar solvents such as diethyl ether (DE) to selectively wash the host solvent DMF. However, by washing the host solvent with a nonpolar solvent, the morphology was improved to some extent; still, there were pinholes in perovskite films with host solvent DMF. This indicates that the removal rate of DMF could not be well-managed while washing with nonpolar solvents. Obviously, the DMSO, as a host solvent for perovskite precursors, forms an adduct with perovskite, and facilitates in improving film morphology by retarding the growth of crystals. Depending on its encouraging performance, Wu et al. [114], in a two-step deposition process, employed DMSO as a precursor solvent for PbI_2_. They described that strong coordination between PbI_2_ and its host solvent DMSO substantially delayed the crystallization process, thereby leading to smooth and dense PbI_2_ films. Pinhole-free better-quality perovskite films with a narrow particle size distribution were obtained after reaction with MAI. Similarly, Seok et al. further applied the solvent engineering approach using DMSO to the FAPbI_3_ system [115]. They reported the intramolecular exchange process which took place between DMSO and FAI while sequentially depositing perovskite films which is expressed as PbI_2_–DMSO+FAI→PbI_2_–FAI + DMSO↑ (removal). High-quality perovskite films with uniform large-size grains are obtained by this indirect reaction between FAI and PbI_2_ as compared to the direct method. However, DMSO has shown to effectively improve the formation of FAPbI_3_ perovskite films by sequential deposition. However, Park et al. disclosed on the basis of infrared spectroscopy measurements [106] that DMSO did not make a stable adduct with FAI, so the approach could not be realistic to one-step deposition of FAPbI_3_. On the same strategy, Yang et al. investigated the influence of Lewis bases on the film growth and resulting film morphology. They fabricated FA based perovskite thin films by using various Lewis bases in a one-step spin-coating technique shown in Figure 5a,b. The photos and SEM images of resulting FAPbI_3_ films are compared (Figure 5c,e for FA-DMSO, and Figure 5d,f for FA-NMP). It can be observed that films formed with DMSO are inhomogeneous and opaque while the films prepared with NMP are homogeneous and semitransparent. They further verified the molecular interaction and adduct forming ability of other Lewis bases DMI, TMU, and NMF with a similar structure to that of NMP and DMSO (Figure 5g). It can also be observed that only DMI formed a stable and uniform adduct film along with NMP. Moreover, NMP used in the precursor can make a stronger coordinate bond with FAI in perovskite solution [109]. It can be confirmed that FAI–PbI_2_–NMP intermediates would exist stably in the host solvent and influence the preparation of more reproducible FAPbI_3_ films as compared to DMI.

Besides NMP, S-donors such as thiourea with stronger basicity demonstrate good potential as a valid Lewis base in perovskite (particularly FAPbI_3_) solutions. Fourier transform infrared spectroscopy results expose the dominant interactions of both FAI and PbI_2_ with thiourea allowing the presence of strong FAI–PbI_2_–thiourea adducts in their host solvents [106]. The perovskite films fabricated from FAI–PbI_2_–(DMSO_1-__x_Theourea_x_) display considerably large size grains and full substrate coverage on the optimized amount of thiourea (x = 0.2); then, the S-donors are more compatible Lewis bases in FA-based perovskites than DMSO.

In the last few years, in addition to S-Donors, N-Donors Lewis-base group has also been studied [116]. Zhang et al. proposed the use of pyridine (Py) additives to fabricate MAPbI_3_ perovskite films at room temperature by a two-step spin coating process [117]. After the spin coating PbI_2,_ the pyridine vapors react with PbI_2_ to form PbI_2_–(Py)_2_ nanostructure. This nanostructured adduct formation is represented by an equation PbI_2_(s) + 2Py(g) ↔ PbI_2_(Py)_2_(s). In the second step, MAI is spin coated on PbI_2_–(Py)_2_; in this step, the perovskite is formed at room temperature by ligand exchange reaction, which is given by the equation PbI_2_(Py)_2_(s) + CH_3_NH_3_(aq)→CH_3_NH_3_PbI_3_(s) + 2Py(aq). It has been reported that the activation energy is considerably decreased by this technique, which facilitated the room temperature fabrication of high-quality perovskite films. The devices fabricated on a rigid substrate delivered a high efficiency of more than 17% and on a flexible substrate more than 14%, which presents the great potential of this approach to obtain low temperature fabricated high-efficiency PSCs.

Additionally, polymers having S, N, and O donor atoms act as Lewis bases; their use as an additive in perovskite precursor solution not only facilitates the perovskite growth process but also suppress the charge defect states at surface and grain boundaries by making an adduct represented as (Pb^2+^–Lewis). Most of all, the presence of hydrophobic polymer at perovskite grains act as a barrier to moisture invasion and increases the stability of PSCs [118]. Gratzel et al. [119] introduced the idea of the polymer-template nucleation and growth (PTNG) by using poly (methyl methacrylate) (PMMA) with antisolvents to influence the nucleation and growth process. They described that PMMA lowers the free energy barrier for nucleation and induces heterogeneous nucleation, which is a high order of magnitude faster than that of homogeneous nucleation [120]. Furthermore, it can effectively control the perovskite crystal growth process by creating an adduct with PbI_2_, which leads to a slow-down of perovskite crystallization to obtain high-quality perovskite films [121]. FTIR spectra have revealed the formation of PMMA–PbI_2_ adducts. The dual role of PMMA in controlling nucleation and crystal formation process enabled to achieve a certified high efficiency of 21% and extended stability. Nevertheless, it has been claimed that the transportation of charge through the perovskite grains is severely affected by the presence of insulating polymers at the grain boundaries. This reduces the performance of PSCs, and π-conjugated polymers are also studied further in this context [122,123,124]. Apart from working the same way to improve the perovskite nucleation and crystallization as insulating polymers do, the π-conjugated Lewis-base polymers have shown a better charge transport through the perovskite film by pi-interactions. Consequently, the photocurrent in polymer-incorporated PSCs has been extraordinarily improved. Nevertheless, the effect of insulating or conjugated polymers on the performance of PSCs is still under discussion [123,124]. It has been described in some reports that only those polymers enable the efficient transportation of charges, which are chemically inert [125]. More in-depth studies are required to elucidate the selection standard of Lewis-base polymers for PSCs.

### 4.3. Solvent Additives

Using a small amount of appropriate solvent additive in the perovskite precursor solution can manipulate the nucleation and thereby facilitate improving the morphology and crystallinity of perovskite thin films. The solvent additive can effectively passivate the point charge defects such as Pb^2+^ in perovskite surface by forming chelation with Pb^2+^ resulting in the improvement in the performance of PSCs [126]. 1, 8-Diiodooctane (DIO) is one of the most extensively used solvents as an additive to control the phase separation in organic and polymer solar cells [127,128]. In the perovskite precursor solution, Jen et al. [110] verified that DIO, being a bidentate halogenated additive, could form the temporary chelation with Pb^2+^ that induces homogeneous nucleation and improve the rate of crystallization, leading to compact and uniform perovskite crystals. Similarly, to investigate the chelation relationship, Eperon et al. introduced a small quantity of HI as an additive in the FAPbI_3_ precursor solution in DMF solvent. They describe that acidic additive increases the solubility of lead halide in the perovskite solution, slows down the perovskite crystallization process, and positively contributes to obtaining pure perovskite phase high-quality thin films [129]. The effect of acid additives on the growth kinetics of perovskite thin films was further investigated by the Sanith group [130]. The colloidal dispersion is formed instead of the pure solution by dissolving perovskite in pure DMF solvent. The obtained perovskite crystals have rod-shaped colloids and poor morphology [131]. The presence of the hydroiodic acids in the precursor solution can lead to the dissolution of the lead poly halide colloids that serve the nucleation sites for growing perovskites in the as-cast thin films. An extended crystal growth period can be observed with the dissolution of large colloids and fewer nucleation sites, leading to larger polycrystalline grain domains. Heterogeneous nucleation controls the growth process, as nucleation occurs on foreign surfaces or through nuclei. The number of desired heterogeneous nucleation sites may be changed by regulating lead poly halide colloids’ concentration in the solution. The perovskite-growing particles would not impose themselves entirely on each other due to the availability of the least number of growing sites, causing pinholes in the active layer. Thus, the lead poly halide colloids’ concentration must be well controlled and optimized to realize pinhole-free perovskite crystals with large-size grains

### 4.4. Solvent Annealing

In this method, a solvent is introduced around the perovskite film, which is soluble (or partially soluble) with the perovskite precursors at the time of thermal annealing. Xiao et al. initiated the work by applying the DMF solvent in a closed environment around a perovskite film during the crystallization by thermal annealing [132].

**Figure 6:**
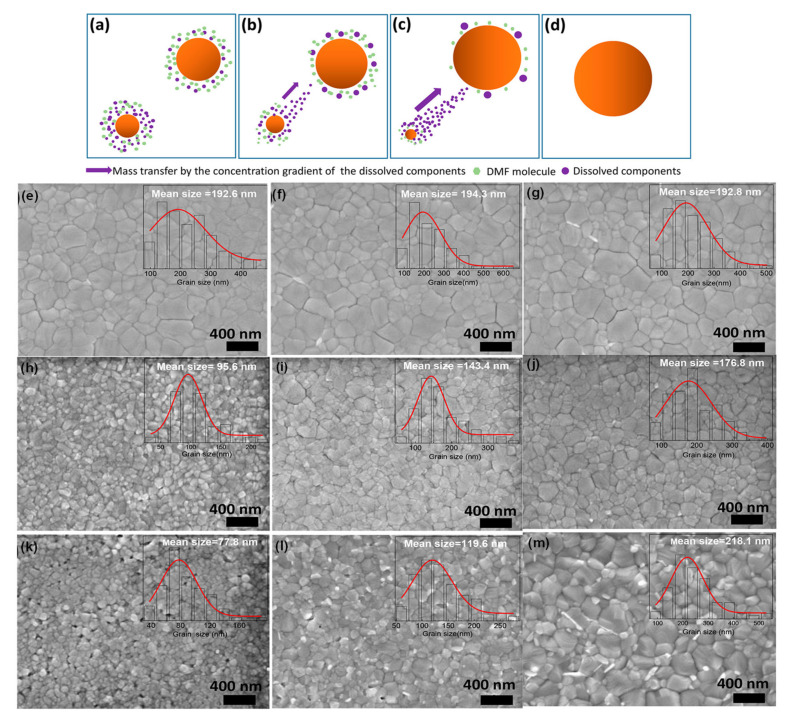
(**a**–**d**) The coarsening mechanism of perovskite grains with lengthened annealing time, as per the Ostwald ripening model: (**a**) initial stage, (**b**) early stage, (**c**) middle stage, (**d**) final stage. (**e**–**g**). SEM images of the perovskite films crystallized at 100 °C for long annealing times after removing the residual solvent: (**e**) 5 min, (**f**) 10 min, and (**g**) 15 min. (**h**–**j**). SEM images of the perovskite films prepared with short annealing times, which still contain some residual solvent: (**h**) 2 s, (**i**) 20 s, and (**j**) 60 s. (**k**–**m**) SEM images of the perovskite films prepared from PbI_2_/DMF solution with 8% DMSO addition, and then annealed at 100 °C for short annealing times: (**k**) 10 s, (**l**) 40 s, and (**m**) 120 s. Reproduced with permission from ref. [134], Copyright 2018, *ACS Applied Energy Materials*.

Here, DMF vapors can produce a moist environment that enables precursor’s components to diffuse longer than conventional thermal annealing methods, thus encouraging grain growth and producing large-sized grains. Subsequent studies are conducted to understand the fundamental processes of how solvent annealing (SA) treatment influenced crystallization [133]. Furthermore, Cao and coworkers reported that the important role played by the solvent during the annealing process. They demonstrated that solvent vapors entrenched in the perovskite precursor films can help in coarsening the perovskite grains [134] as shown in Figure 6a–d. However, owing to the rapid escaping of the solvent at high temperatures, the small grains’ dissolution cannot happen. The long annealing time (from 5–15 min) at 100 °C ensure the escape of DMF solvent, and the absence of DMF reduces the Ostwald ripening process, leading to very little change in grain size and morphology (Figure 6e–g). They also characterized the morphology of perovskite annealed for a short time. They found that extended thermal annealing can increase the grain size (Figure 6h–j). The increasing size grain was attributed to the happening of the solvent-mediated Oswald ripening process during prolonged annealing. They further prepared perovskite films by using mixed (DMF and DMSO) host solvents for PbI_2_. The prominent role of Oswald ripening on the perovskite grain size growth is clearly observed (see Figure 6k–m) by mixing the optimized amount of DMSO, which is a strong base and has a high boiling point than the other component (DMF) of the host solvent. These results endorse the residual solvent’s important function in the occurrence of the Ostwald ripening process with the SA technique. Lastly, it was described that the most frequently used solvents (such as DMSO and DMF) have good solubility for both PbI_2_ and MAI and definitely can damage the perovskite film in the solvent annealing treatment under the uncontrolled vapor pressure [135]. To address the problem, alcohol vapors are engaged for the solvent annealing process, which has selective solubility for MAI over PbI_2_. Vapors of different alcohol having different polarities are reported to finely modulate the reaction rate of perovskite precursors and the growth of crystals. This technique helped to remove pinholes and passivated the defects, thereby leading to obtaining better quality perovskite films [135].

### 4.5. Nonstoichiometric Composition

During thermal annealing, because of the rapid depletion of the reaction medium (evaporation of solvents), the uniform growth of perovskite grains is relatively challenging. Extended heat treatment can lead to decaying the perovskite films due to the volatile nature of organic components. Therefore, the unequal molar concentration of perovskite components in solution has been found to help in suppressing the recombination at the grain boundaries (GBs), thereby allowing the smooth transfer of charge carriers. Yang et al. [136] have suggested employing a nonstoichiometric perovskite solution with excessive MAI to complement the volatile organic component during the grain formation at a higher temperature. With the use of nonstoichiometric perovskite solution’s large area, smooth perovskite films with improved morphology are achieved. A remarkable efficiency of 15.3% is obtained for the device with an active area of 1.2 cm^2^. Numerous postfabrication procedures have been established to encourage grain growth. Control of supersaturation is extensively implemented to manage the rate of nucleation, which enables to regulate the grain size of perovskite crystal. In 2014, Im et al. described that the rate of nucleation could be regulated by changing the concentration of CH_3_NH_3_I solution in two-step deposition [137]. In this method, spin-coated PbI_2_ films react in the solution with the MAI, where CH_3_NH_3_PbI_3_ is supersaturated if the MAI concentration becomes higher than the 0.02 m equilibrium concentration. It is obvious that nucleation with 0.063 m MAI solution becomes much faster due to increased MAPbI_3_ supersaturation in the precursor solution, producing a denser film with smaller size grains than the film based on 0.038 m MAI solution. Following a similar strategy, in 2016, Kim et al. [138,139] observed that a small quantity of excess PbI_2_ in precursors could impressively enhance the photovoltaic performance of PSCs devices. They proposed a nucleation and growth mechanism for PbI_2_ employing high-resolution transmission electron microscopy and noticed that apparently, a small extra amount of PbI_2_ could improve perovskite crystallinity, and thereby, impede ion migrations and decrease recombination. Thus, the addition of an excess amount of PbI_2_ in the absorber is commonly used by numerous groups as an effective process to improve the performance PSCs. Following the nonstoichiometric composition, Chen et al. showed impressive enhancement in PCE, 0.66% to 12%, by compositional/structural conversion of extra CH_3_NH_3_PbI_3_ to PbI_2_ occurred mostly at the GBs of the film during thermal annealing. This improvement in efficiency is attributed to the passivation of surface defects [140]. On the other hand, PbI_2_ can also be merged into perovskite absorbers by the addition of excess PbI_2_ to the precursor solution, which has been suggested as a means of decreasing J–V hysteresis by hindering ionic migration [139]. In further studies, Nazeeruddin et al. reported that unreacted PbI_2_ remaining as a secondary phase during dip coating can decrease charge carrier recombination at the perovskite/cathode interface, thereby improving device performance [141].

## 5. Scalable Fabrication of Perovskite Solar Cells

The fabrication of a smooth and pinhole-free large-area perovskite thin film is critically important to realize PSCs’ commercial application. Until now, the understanding of crystal growth mechanisms and precursor solution chemistry has enabled remarkable progress established on several scalable deposition techniques. This section summarized the significant improvement in the fabrication of large-area perovskite films and PSCs with scalable deposition methods.

### 5.1. Blade Coating

Blade coating is the most reliable, scalable, and widely used technique adopted in solution-processed perovskite solar cells. Minimum material waste, easy to operate, and compatible with roll-to-roll manufacturing makes it the most promising deposition technique [37]. The thickness of the blade coated film is generally determined by several internal and external factors.

Many research reports reveal that the blade coating produces a uniform wet film from the precursor solution by one-step deposition [79,142]. The applied external processing strategies largely control the quality of the crystallized solid films; therefore, control of the crystallization is critical to obtaining smooth and high-quality thin films during blade deposition. The comparatively slow solvent evaporation rate of freshly blade coated films facilitates larger crystals to grow; however, to obtain compact films with the natural drying process is challenging [143]. Temperature and atmospheric environments during annealing have been found to be crucial to obtain the films with preferred morphology [80]. For instance, both the gas blowing [91] and preheated high-temperature substrate [78] are described as promising approaches to control the perovskite nucleation and crystal, thereby resulting in high-quality perovskite films with decent PCEs of more than 20%. Huang et al. [79] reported the blade coating of perovskite films on a preheated substrate. The substrate’s temperature as high as ≈70–145 °C created a fast supersaturation of the precursor in the solution, leading to the rapid and uniform perovskite crystal growth. Low substrate temperature causes crystallization of intermediate phases with a needle-like shape that obstructs the films’ complete coverage. A coffee-ring-like structure with broad domains was detected after the solvent’s evaporation on the preheated substrate, which is attributed to the convection of the solution.

**Figure 7:**
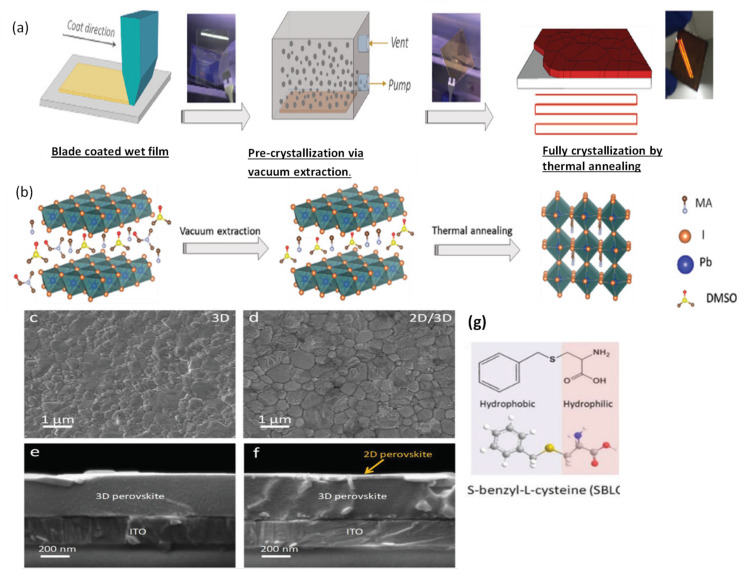
(**a**) Represents the one-step deposition and vacuum-assisted crystallization of a blade coated perovskite film. (**b**) Printed precursor colloids then vacuum extraction to create an intermediate film by removing solvent from printed wet film and finally the fully crystallized perovskite film after thermal annealing. Reproduced with permission from ref. [37], Copyright 2019, *Advanced Science*. (**c**,**d**) SEM images of the surface of 3D perovskite film and 2D/3D perovskite film. (**e**,**f**) Cross-sectional SEM images of the 3D perovskite film and 2D/3D perovskite film. The 3D and 2D/3D perovskite films are fabricated by blade coating of MAPbI_3_ precursor without and with addition of 5 mg mL^−1^ SBLC, respectively. (**g**) Molecular structure of the SBLC additive. Reproduced with permission from ref. [145], Copyright 2020, *Advanced Energy Materials*.

Alternatively, room temperature blade coating and vacuum-assisted crystallization approaches have been reported (see Figure 7a,b), yielding high-quality, pinhole-free perovskite thin films [37]. Using this strategy, Qiu et al. [144] reported the high electronic quality of the environment-friendly, economical biopolymer (poly-l-lysine) passivated perovskite solar cells, yielding an obvious increase in V_OC_ by up to 100 mV. They obtained a high PCE of 19.45% in their blade-coated device. Similarly, following the same crystallization protocol, Hu et al. [145] proposed a 2D/3D mixed perovskite by blade-coating (see Figure 7c,d). First, the 3D MAPbI_3_ bulk perovskite is blade coated, followed by a thin layered 2D perovskite, which is formed by the judicially selected organic cation (S-benzyl-_L_-cysteine) as a spacer molecule (Figure 7g). The 2D/3D layered heterostructure makes as a well-defined interface and can be observed in microscopy imaging (shown in Figure 7e,f). It is reported that the two functional groups’ carboxyl and amine present in the spacing molecule of the 2D perovskite facilitates in passivating the surface defects of the 3D perovskite. Consequently, a noticeable increase in open-circuit voltage (V_OC_) up to 100 mV, and a high efficiency of 20.14% is delivered by the champion device.

Blade coating has been successfully applied for the deposition of thin films for scalable PSCs. Huang et al. [78] used small amounts of surfactant (L-α-Phosphatidylcholine) that vividly changed the fluid’s drying dynamics and increase the adhesion of the perovskite ink on the surface of the nonwetting charge transport layer. The additives facilitated the blade coating of uniform and pinhole-free perovskite films with root-mean-square roughness of 14.5 nm over 1 cm. Furthermore, the surfactant additive passivated the surface defects, resulting in PCE of over 20% in small area devices, 15.3%, and 14.6% in large aperture areas of 33.0 cm^2^ and 57.2 cm^2^, respectively.

Similarly, Zhu et al. [142] described the perovskite module’s fabrication via the doctor blading with a total area of 12.6 cm^2^ and an active area of 11.09 cm^2^ yielding a PCE of 14.06% from the reverse scan. The little amount of additive MACl was mixed in MAPbI_3_ perovskite precursor, facilitated crystallization by increasing the rate of grain growth, and shortened the annealing time to obtain better-quality perovskite films. Congping Li et al. [146] used monoammonium porphyrin (ZnP) as a surfactant additive for blade-coating stable large-area perovskite solar cells. They described that the surfactant additive could efficiently passivate the surface defects by firmly attaching to the perovskite surface. Furthermore, the ZnP additive provides a perfect molecular encapsulation to the perovskite surface, thereby avoiding volatile cations to escape. They successfully blade coated smooth and pinhole-free high-quality large area (16 cm^2^) perovskite films. A high PCE of 18.3% with a large area of 1.96 cm^2^ was remarkably achieved, while the PCE for the small-area (0.1 cm^2^) solar cell was up to 20.5%. These results indicate that the addition of ZnP effectively passivated the surface defects, controlled the perovskite grain growth, and facilitated pinhole-free fabrication of perovskite films. Guo et al. [37] developed a controlled crystallization protocol that fulfills the requirement of low-temperature deposition of precursor films, which is extremely needed for large-scale perovskite thin-film fabrication (as shown in Figure 7a). To assess the promising scalability to fabricate large-area PSCs by blade coating method, they fabricated a large-area module with an active area of 10.08 cm^2^. Their best module having four cells interconnected monolithically in series, displayed a high PCE of up to 15.38%. Notably, the insignificant V_OC_ losses (22.5 mV) for each sub cell show that high-quality 2D/3D heterojunction perovskite films can be deposited on a large scale by one-step blade-coating technology. They obtained V_OC_ of 1.17 V, which is the highest value ever reported in perovskite modules for subcells. Furthermore, it is reported that the lower FF of the module is probably because of the higher series resistance. This drawback is possibly associated with the patterning procedure rather than the perovskite film [145].

In short, currently, blade coating is the most efficient and economy scalable coating technique that produces uniform films for PSCs. However, the disadvantage is that only the perovskite active layers of the most efficient devices were coated by blading or other scalable methods, and the perovskite layer was blade coated in a glovebox under a controlled atmosphere. Therefore, it is of highly required to design suitable perovskite ink chemistries that can be processable in ambient environment and the improvement in coating technique to fully blade coat the efficient PSCs.

### 5.2. Slot-Die Coating

The slot-die coating is another extensively used scalable organic and perovskite thin-film fabrication technique. In this technique, the precursor is regularly delivered from the coating head towards the substrate [20]; continuous meniscus liquid edge forms in the gap between the substrate and coating head, which is used to derive the spread of liquid film across the substrate. Fast and accurate coating applied to the slot die is useful for large-scale manufacturing with high output [147]. During the coating, the wet film thickness can be estimated by supplied ink quantity and theoretical calculation to control all the processing parameters. In this technique, the morphology of the crystallized perovskite film predominantly relies on the external strategies during the solidification process and the solvent extraction rate.

**Figure 8:**
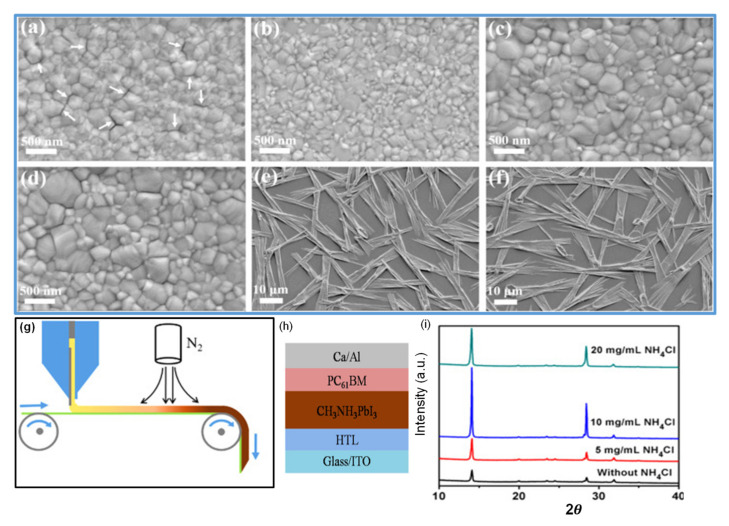
(**a**–**f**) SEM images for CH_3_NH_3_PbI_3_ films prepared using different conditions: (**a**) without NH4Cl additive; (**b**) 5 mg/mL NH_4_Cl additive; (**c**) 10 mg/mL NH4Cl additive; (**d**) 20 mg/mL NH4Cl additive; (**e**) spin coating, without NH_4_Cl additive; (**f**) spin coating, 10 mg/mL NH_4_Cl additive. (**g**) Roll-to-roll continuous preparation of CH_3_NH_3_PbI_3_ films. (**h**) Device structure of perovskite solar cells. (**i**) The XRD results. Reproduced with permission from ref. [149], Copyright 2018, *Elsevier Nano Energy*.

Cotella et al. [148] investigated the influence of air-knife blowing and substrate temperature on the perovskite film formation during the slot-die coating. Preheating the substrate to 65 °C and air-knife blowing during slot-die coating can increase the drying dynamics and accelerate the crystal growth process. Finally, wet perovskite films can be quickly dried, producing a considerably fast precursor supersaturation, leading to heterogeneous nucleation on the surface of the heated substrate. Chemical additives are also used in the perovskite solutions to enhance the degree of crystallinity of solid perovskite films. Zuo et al. [149] demonstrated a simplistic blowing-assisted drop-casting (BADC) method to fabricate CH_3_NH_3_PbI_3_ films for perovskite solar cells (see Figure 8g). The effects of the NH_4_Cl additive on the morphology of CH_3_NH_3_PbI_3_ films have been investigated by the SEM images. The perovskite films prepared without additives have shown some cracks and pinholes, which result in device shunt resistance (see Figure 8a). Cracks and pinholes disappeared by adding 5 mg mL^−1^ NH_4_Cl additive (see Figure 8b). The films prepared with 10 mg mL^−1^ NH_4_Cl additive demonstrate a much bigger grain size than without and with 5 mg mL^−1^ additive (Figure 8c). The grain size is similar in films formed with 20 mg mL^−1^ NH_4_Cl additive, but a smaller crystallite size is observed (Figure 8d). They also attempted spin coating of perovskite films under the same condition without and with 10 mg mL^−1^ NH_4_Cl additive (see Figure 8e,f). Poor morphology with huge pinholes can be observed in the films. As a result, the optimized 10 mg mL^−1^ NH_4_Cl additive, to better the crystallinity (Figure 8i) of the perovskite film, is utilized. They also prepared PSCs in the air with a structure of ITO/m-PEDOT:PSS/CH_3_NH_3_PbI_3_/PCBM/Ca/Al (see Figure 8h) and obtained a maximum PCE of 19.48%. The optimized design is then effectively applied to slot-die coating on a glass substrate and next to roll-to-roll on a flexible substrate, giving the record PCEs of 15.57% and 11.16%, respectively.

Nevertheless, the slot-die coating also has some drawbacks, such as the wastage of a sufficiently large quantity of perovskite ink to fill up the ink reservoir and the supply pipes during device fabrication. Therefore, this technique is much more expensive for laboratory applications compared to the blade coating. As a result, the slot-die technology is not employed frequently in research labs, and the slot-die coated devices have much lower PCE than blade coating PSCs.

**Figure 9:**
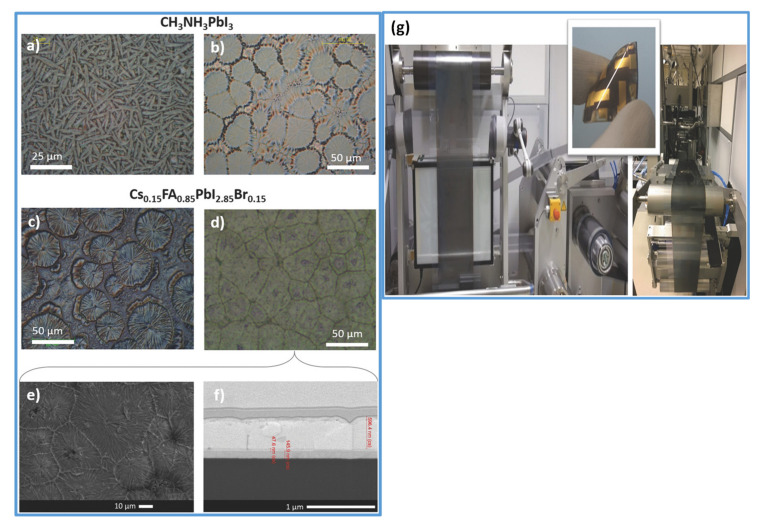
(**a**,**b**) The microstructures of the CH_3_NH_3_PbI_3_ layer dried at 140 °C with slow and fast temperature ramping, respectively. (**c**,**d**) Cs_0.15_FA_0.85_PbI_2.85_Br_0.15_ layer dried with slow and fast temperature ramping, respectively. (**e**) The SEM image of perovskite (Cs_0.15_FA_0.85_PbI_2.85_Br_0.15_) layer. (**f**) Cross-sectional FIB–SEM image of SnO_2_ and perovskite layers R2R coated on PET/ITO substrates, the perovskite layer Cs_0.15_FA_0.85_ PbI_2.85_Br_0.15_ dried with fast temperature ramping (corresponding to the images **d**,**e**). (**g**) Photograph images of the roll-to-roll prepared perovskite layer (Cs_0.15_FA_0.85_PbI_2.85_Br_0.15_) and an example of the manufactured flexible devices. Reproduced with permission from ref. [155], Copyright 2018, *Advance Energy Materials*.

The slot-die coating process has been proposed as a roll-to-roll compatible process for the manufacturing of PSCs in recent years individually by Hwang et al. in 2015 [90] (Ciro et al. [150], Cotella et al. [148], and Qin et al. [151] in 2017), (Zuo et al., Di Giacomo et al., and Kim et al. in 2018) [149,152,153]. Excellent performance has been reported so far on slot-die-coated PSCs. However, this has been demonstrated mostly for small area devices with small aperture masks for testing. Several times the slot-die coating technique has applied for the scalable fabrication of perovskite solar cells. The first fully slot-die-coated perovskite module with an active area of 40 cm^2^ was reported in 2015, by Hwang et al. [90]. It was described that the slot-die coating of MAI at room temperature produced a thin and incomplete perovskite layer. Therefore, to improve the quality of perovskite, they heated PbI_2_ coated substrate, which accelerated the perovskite conversion reaction. Consequently, dense films with cubic shape crystals were obtained, leading to improving the PCE from 4.20% to 11.96% at an optimized substrate temperature of 70 °C [90]. Based on the fact that the fast nucleation encourages the formation of dense, uniform, and compact perovskite films, therefore, adding the PbCl_2_ in perovskite further increases the Ostwald ripening of the small grains results in films with large size grains. Later in 2018, Lee et al. [154] fabricated a PCE of 13.3% in a small cell and 8.3% in a 10 cm^2^ submodule using mixed Pb precursors. Following this, Giacomo et al. [152] also employed the mixed Pb perovskite precursors solution and fast crystallization kinetics to fabricate large-area PSCs using a slot die coating. Perovskite films deposited with slot die exhibited a superior crystallinity with a larger grain size than the spin-coated films. They fabricated perovskite thin films and the hole-transporting (spiro-MeOTAD) layers for submodules (168.75) cm^2^ and obtained the PCE of more than 10%. Similarly, for upscaling the perovskite solar cells, in 2018, Galagan et al. [155] demonstrated the coating of perovskite thin films by roll-to-roll slot-die technique (Figure 9g) which resulted in a uniform, transparent layer with a thickness of about 45 nm on a flexible substrate with a width of 30 cm and the web speed of 3–5 m min^−1^ for efficient, flexible solar cells. The average stabilized PCE achieved for the devices made on different areas of the foil is 12%, with the best value of 13.5%.

A mixed-cation mixed-halide perovskite (Cs_0.15_FA_0.85_PbI_2.85_ Br_0.15_) by applying similar solvent composition and drying settings has also been demonstrated. As estimated, the crystallization pattern of the roll-to-roll manufactured Cs_0.15_FA_0.85_PbI_2.85_Br_0.15_ layer is different from the CH_3_NH_3_PbI_3_ layers. The microstructures of Cs_0.15_FA_0.85_ PbI_2.85_Br_0.15_ thin films with both slow and fast temperature ramp-up enable plate-like crystals (see Figure 9c,d) and there is no inclination to the formation of needle-like crystals (see Figure 9a,b), which has been observed in the CH_3_NH_3_PbI_3_ composition. The SEM images of Cs_0.15_FA_0.85_ PbI_2.85_Br_0.15_ thin films with fast temperature ramp-up and cross-sectional FIB-SEM images of SnO_2_ and perovskite layers R2R coated on PET/ITO substrates, with fast temperature ramping are shown in Figure 9e,f. Furthermore, it looks as the fast temperature ramp-up has a positive influence on the crystallization process: the grains of crystal are found to be compact, resulting in a uniform layer.

In short, it is scalable and a continuous production technology. Fully slot-die-coated (except metal electrode) PSCs fabricated with low PCE up to 9%. Therefore, full comprehension of the crystal growth mechanism and formulation of new solutions engineering might be essential to improve PCE and further move towards continuous production. Additionally, revising the several processing factors, including solution dynamics, fluid dynamics, and solution delivery may help produce the most efficient PSCs by this coating method.

### 5.3. Spray Coating

Spray coating is a useful low-temperature thin film deposition technology that provides a continuous, high coating rate suitable for different substrates. It has been used to deposit organic photovoltaic (OPV) films, oxide thin films, and painting [156]. Its process consists of four consecutive stages: the formation of droplets, the transfer of the droplets towards a substrate, the coalescence of the droplets into a wet film, and the last step is the annealing process [157,158]. The droplets are generated through a nozzle from the coating solution. The control of the droplets’ size and uniformity is critically important to obtain a uniform coating across a surface. This technique also attracts great attention in the fabrication of perovskite thin films [159]. However, less perovskite studies have been reported using the spray coating method, perhaps because of PSCs’ instability in the ambient atmosphere.

In the last few years, significant efforts have been made to control perovskite crystallization during the spray coating of PSCs. In 2014, Barrows et al. fabricated the first planar structured PSCs, using a spray coating technique. In this study, spray solvent volatility, the post-deposition heating time, the effect of substrate temperature on perovskite film, and surface coverage of the perovskite were studied. The grain size and the substrate coverage appeared to be different with different substrate temperatures because of changes in nucleation and crystallization rates. Through the optimization, the film with the highest coverage of 85% could be obtained at the substrate temperature of 75 °C, which resulted in a PCE of 11% [160].

Das et al. highlighted that the controlled substrate temperature is essential to improve the resulting perovskite films’ coverage. They displayed the option of using the spray coating method for roll-to-roll device fabrication. They demonstrated perovskite solar cells on a flexible substrate and obtained efficiency up to 7.3% [159]. In 2016, Ishihara et al. applied a two-solvent system to spray coat the PSCs. They added a small surface tension surfactant in solvents to reduce the liquid surface tension. In the spray-coating process, they observed the fluid dynamics of droplets. The volume ratio of solvents (NMP and DMF) was altered and optimized to produce a local surface tension gradient. So, droplets can spread and form a continuous wet film on the substrate to attain perovskite films with high surface coverage. Thus, PCE of the devices made through the two-solvent system reaches 14.2%, which is one-and-a-half times that prepared with the one-solvent spray pyrolysis [161].

The two-step deposition strategy has proven to be more feasible, as perovskite films are somewhat less affected by their surrounding atmosphere. Sequential spray-coated perovskite films have shown better photovoltaic performance and substrate surface coverage [162]. Huang et al. described the sequential spray coating of PbI_2_ and MAI solution [163]. Postannealing induced the interdiffusion of two-step deposited precursor layers, which then converted to MAPbI_3_ perovskite films. It was described that the morphology of the PbI_2_ layer could be modified by controlling the substrate temperature during the spin-coating. In the spray coating process, the control of substrate temperature while the film is being fabricated was comparatively easy. By systematically optimizing the fabrication process, the MAPbI_3_ films with full surface coverage were successfully deposited. The resulting MAPbI_3_ films displayed an improved PL lifetime compared with those formed by a sequential spin coating process, resulting in higher performance of the devices than those based on the spin coating process. The stabilized PCE of 15.4% can be achieved with an active area of 0.1 cm^2^ and 13.09% with an active area of 1 cm^2^.

Many attempts have been made to get better PCEs in large-area solar cells using a spray coating method. The relatively better PCE (of an average 13.7% in 1 cm^2^ active area) in spray-coated MAPbI_3_ devices is achieved using the mega sonic-spray method in which a 1.7-MHz mega sonic nebulizer is utilized [164]. In this method, mist droplets are formed, which are narrower and smaller than those achieved in conventional ultrasonic spraying and result in uniform grain size. The spray coating of FAPbI_3_-based perovskite is less studied compared with MAPbI_3_. Based on this technique, an FA-base with mixed halides perovskite film on a large substrate was fabricated by applying spray coating and chemical-vapor-transport deposition. First, they coated the PbI_2_ by the spraying technique followed by the vaporized mixed organic halides to react with the PbI_2_ film using the chemical-vapor-transport method. PSC made using this feasible technique showed a better efficiency of 14.7% [165]. In another study, Liu et al. [166] used a scalable, automated ultrasonic one-step solution process spray deposition method for the fabrication of large-area perovskite CH_3_NH_3_PbI_3_ films. Their device with an active area of 1 cm^2^ displayed a better photovoltaic performance and good moisture stability. A PCE of 12.30% was achieved by optimizing perovskite composition. By following the same optimized coating process, they fabricated the large-area PSCs and obtained 10.18% efficiency for the device area of 2 cm^2^ and 7.01% in the 3 cm^2^ device area. Tait et al. [167] also utilized the same optimized ultrasonic spray coating method and formulated the precursor composition. The ratio of MAI, MABr, PbI_2_, PbCl_2_, and PbAc_2_ in the precursor solution was judicially optimized and finally improved the perovskite crystals’ morphology. They achieved the efficiency of 13.7% for an area of 0.13 cm^2^, and 10.4% PCE for the device with an active area of 3.8 cm^2^.

**Figure 10:**
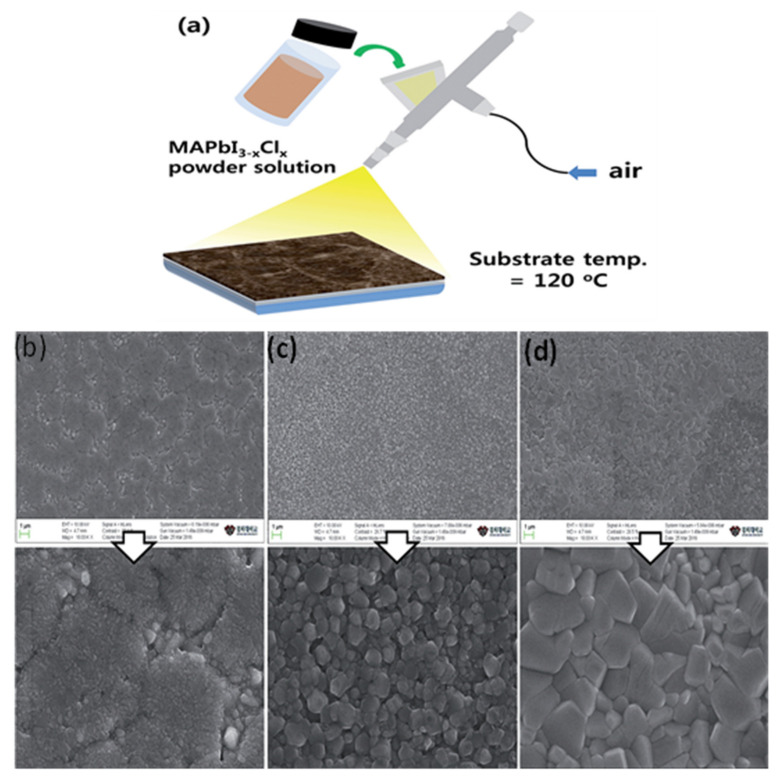
(**a**) Represents the fabrication of the MAPbI_3−*x*_Cl*_x_* mixed perovskite film by spray coating through a solution of MAPbI_3−*x*_Cl*_x_* mixed halide powder. (**b**–**d**) SEM images of MAPbI_3−*x*_Cl*_x_* mixed halide perovskite films at spraying times of 30 s (**b**), 60 s (**c**), and 90 s (**d**). Reproduced with permission from ref. [168], Copyright 2016, *Journal of Materials Chemistry A*.

In 2016, Lee et al. [168] demonstrated spray-coating of mixed halide MAPbI_3_-_x_Cl_x_ perovskite on a preheated substrate and achieved large-grain-sized perovskite films by optimizing the inward flux (F_in_) and the outward flux (F_out_) (see Figure 10a). They used two solvents, the high boiling point GBL and the low boiling point DMF solvent, in a volume ratio of 8:2 to get the large size (an average of 1.5 μm) perovskite crystals.The perovskite crystal grains continue their growth as the spraying time increased to 30 s (Figure 10b), 60 s (Figure 10c), and 90 s (Figure 10d). In the initial stage, the formed perovskite grains are small. Then, on the replenishment of the MAPbI_3_-_x_Cl_x_ solution, in accordance with Ostwald ripening, the small grains are redissolved and recrystallized into large grains. The better PCE of 15.5% is achieved for the submodule. In subunit cells, its PCE reaches up to 18.3%, which is the highest efficiency until now. These results indicate that the spray-coating method has great potential for achieving economical, large-area, and efficient PSCs.

**Figure 11:**
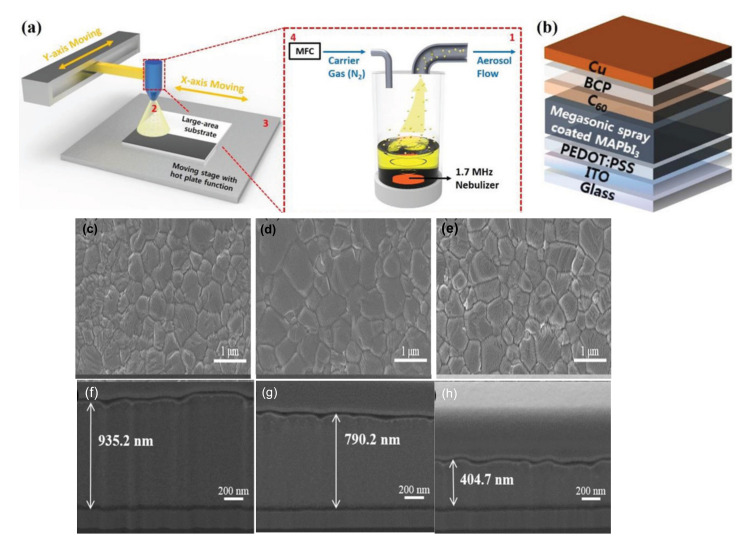
(**a**) Represents the megasonic spray coating process. (**b**) Represents the inverted planar MAPbI_3_ PSC fabricated by the megasonic spray coating system. (**c**–**h**) Surface and FIB-cross-section SEM images of the perovskite film coated on glass/ITO/PEDOT:PSS with scan speed: (**c**,**f**) 10 mm s^−1^, (**d**,**g**) 15 mm s^−1^, and (**e**,**h**) 20 mm s^−1^. The films were annealed at 120 °C for 1 min with DMSO vapor after coating and for 4 min in air. Reproduced with permission from ref. [164], Copyright 2019, *Small*.

In 2019, Park et al. [164] demonstrated continuous spray coating of CH_3_NH_3_PbI_3_ films employing a 1.7 MHz megasonic nebulizer (see Figure 11a). They reported large-area 7.5 × 7.5 cm^2^ planar architecture perovskite devices (Figure 11b) with an active area of the single-cell up to 1 cm^2^. The device delivered a top high efficiency of 14.2% and an average PCE of 13.7%, which is the highest PCE ever reported in spray-coated PSCs. They have demonstrated that the morphology of the perovskite surface slightly depends upon the scan speed. Almost similar perovskite film morphology is observed at a scan speed of 10, 15, and 20 mms^−1^ (see Figure 11c–e). The grain size varies only with different scan speeds (see Figure 11f–h). The largest grain size (790 nm) is observed at a scan speed of 15 mms^−1^ (see Figure 11g). High-quality perovskite active layer with large-scale grain has been achieved by controlling the reaction temperature and the flowing rate of the CH_3_NH_3_PbI_3_ precursor solution [164].

In short, spray coating is a scalable low-temperature thin-film fabrication technology. After blade coating, it is the most efficient large-area coating technique. However, mostly single-step spraying deposited perovskite film morphology is not uniform, having dendritic, or incomplete substrate coverage, which causes the poor PV performance of PSCs than those fabricated by other scalable coating techniques. Enormous shunt resistance lowers all the performance parameters. Therefore, optimizing the substrate temperature, spraying speed, the distance between the substrate and nozzle, the perovskite precursors composition, the precursor host solvents, and additives improves the perovskite film morphology and the device performance. The wastage of a large quantity of the perovskite solution is a big challenge for this method’s feasibility to become a promising scalable technology. In the future, it could be expected, with more devoted hard works, to apprehend a high flux spray pyrolysis process that would enable economical for perovskite solar modules.

### 5.4. Ink-Jet Printing

An inkjet printing technology enables fast and material-conserving coating of thin films for the large-scale fabrication of photovoltaic thin films with the ability to produce preferred patterns. However, the delicate control of crystallization of the deposited wet films and solution properties is a basic requirement to improve printing accuracy [169]. In this printing technology, control of the drop size is essential to obtain uniform thickness film. The ink properties play a vital role in ensuring the formation of stable droplets at the nozzle. Furthermore, the quality of the fabricated film predominantly relies on the scattering of drops, coalescence, crystallization, and homogeneity of the film during annealing. The above factors are basically determined by the ink droplet’s interaction with the substrate, for example, the contact angle and surface roughness, etc. [18]. The amusing achievement significantly encourages the use of this processing technique in perovskite devices.

**Figure 12:**
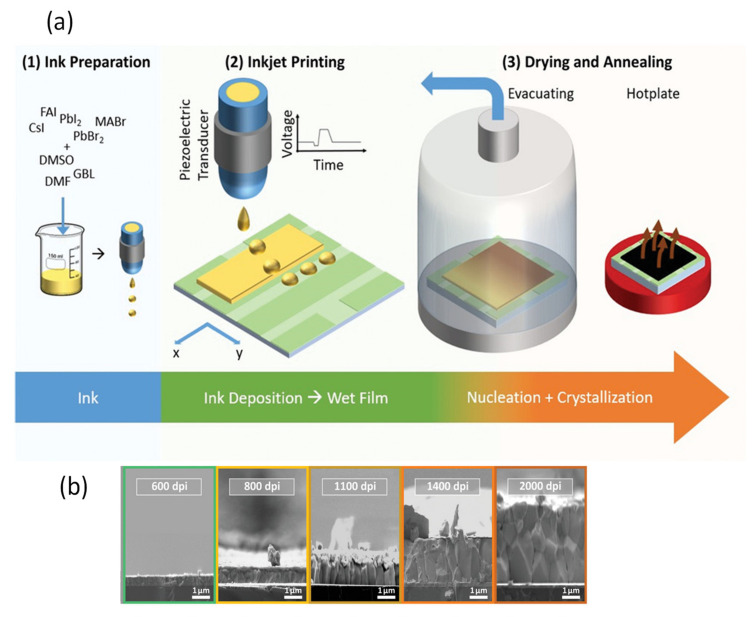
(**a**) Represents the important steps involved in inkjet printing of triple-cation multicrystalline perovskite (TCP) thin films: (1) ink preparation, (2) inkjet printing, (3) drying and annealing: The printed TCP wet film is transferred to a vacuum chamber to accelerate the rate of removal of the solvents to induce nucleation of the perovskite thin film. (**b**) SEM images of cross sections of PSCs with inkjet-printed perovskite films with different resolutions. Reproduced with permission from ref. [174], Copyright 2020, *Advanced Energy Materials*.

In 2014, Yang et al. first fabricated metal-electrode-free PSCs by inkjet printer [170]. For the top electrode, carbon is applied, which is inert to iodide in perovskite. Due to the synergistic effects of carbon incorporation, high efficiency of 11.6% was achieved in small area devices. In a subsequent study, Song et al. [171] fabricated perovskite precursor films by using one-step inkjet printing. The temperatures of the substrate again can strongly influence the film-forming process, and the substrate heated at 50 °C quickly removed the solvents for uniform film thickness. In 2016, Mathies et al. [172] demonstrated an additional vacuum annealing step after precursor film deposition. They point out that the vacuum annealing method could improve crystallinity, thereby obtaining compact and uniform perovskite film with better morphology. The PSCs fabricated using a vacuum-annealed process and optimizing the distance between the printing drops and the maximum number of printed sublayers delivered a high PCE of 11.3%. In 2018, Mathies et al. [173] also extended the technology to mix triple cations PSCs. The devices fabricated deliver much higher efficiency than the mono cation-based PSCs, which was the maximum of 15.3% in the best device. The printed triple cation-based PSCs also significantly outperformed MAPbI_3_-based PSCs in both extended stability and efficiency. The PCE of triple cation-based PSCs decreased only 10% at 80 °C temperature for around 120 min, while MAPbI_3_-based devices were nearly totally degraded. In 2020, Eggers et al. [174] demonstrated good-quality inkjet-printed triple-cation perovskite thin films with excellent thicknesses of >1 µm. The thickness of the inkjet-printed absorber layer increases with printing resolution (see Figure 12b). The range of perovskite thin films is around 400 nm (600 dpi) up to almost 4 µm (2000 dpi). They demonstrate maximum high PCEs of more than 21% and greater than 18% stabilized power output efficiencies for their inkjet-printed PSCs. For crystallization, the printed wet films can be moved physically to a close vacuum chamber (see Figure 12a). They evacuated the vacuum chamber for a few minutes. When the pressure decreased to 5 × 10^−2^ mbar, it declined the boiling point of the solvents, and increased the evaporation rate of the solvents. On the basis of their boiling point, first DMF started to evaporate, then followed by DMSO and GBL. When the removal of solvents is completed, then the crystallization of wet film started. Next, the chamber was gently vented with ambient air, and the samples then can be annealed on a hotplate under ambient air.

It is also revealed that perovskite thin films fabricated by inkjet printing techniques exhibit solute agglomeration and rod-shapes if printed from high ratio DMF or high ratio DMSO, respectively. Li et al. obtained the balance between the ink solvent’s evaporation rate by mixing DMF and DMSO in an equal volume ratio. They successfully obtained the compact perovskite crystals with a grain size greater than 2 µm by optimizing the droplet’s physical properties. Due to the good crystallinity of the absorber layer, the fabricated devices demonstrated high efficiency of 18.6% with an active area of 0.04 cm^2^ [175].

**Figure 13:**
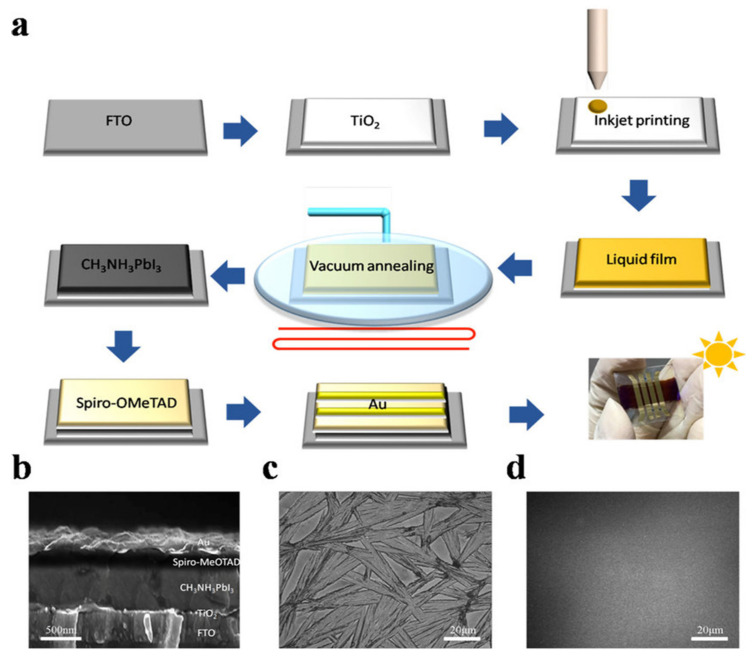
(**a**) Represents the fabrication of PSC through inkjet printing. (**b**) Cross-sectional SEM of a complete photovoltaic device. (**c**,**d**) The optical images of inkjet-printed MAPbI_3_ thin film by conventional annealing and vacuum-assisted thermal annealing. Reproduced with permission from ref. [176], Copyright 2018, *Solar RRL*.

In 2018, Xing et al. [176] produced inkjet-printed good-quality perovskite films through optimizing the solvent composition and vacuum-assisted thermal annealing posttreatment method. Inkjet-printed thin film growth techniques and basic steps involved in device fabrication are shown in Figure 13a. Noncontinuous perovskite dendrites are observed in thin films after conventional annealing as shown in Figure 13c. However, with the vacuum-assisted thermal annealing posttreatment strategy, extremely uniform and dense films are obtained as shown in Figure 13d. The planner heterojunction MAPbI_3_ PSCs have been fabricated (as shown in Figure 13b) and by the synergetic effect of these two techniques, the PCE previously reported as ≤12.3% can be further enhanced to 17.04% for devices with area 0.04 cm^2^ and 13.3% for large area 4.0 cm^2^. The addition of NH_4_Cl as an additive into the perovskite precursor solution is found to stimulate the growth of perovskite crystals with desired orientation under ambient conditions [177]. Upon exposure to moderate humidity (≈RH 35%), the intermediate phases can be converted slowly to perovskite crystals with preferred orientation. Liu et al. [178] fabricated self-assembled silane layers to decrease the recombination of the charge carriers at the interfaces, which improves the V_OC_ of the devices. Hu et al. [179] also successfully demonstrated a 10 cm × 10 cm module delivering a PCE of 10.4%.

In 2017, Grancini et al. [180] demonstrated a 2D−3D interface engineering method for hybrid perovskite film formation to enhance solar device performance and stability. Their 10 cm × 10 cm module with an active area of 47.6 cm^2^ delivers a PCE of 10.10% with one-year comprehensive stability. Remarkably, the module showed no decrease in PCE after 12 h. Some other groups have also used this printing process to fabricate the solar cells and modules. However, their photovoltaic efficiency is still comparatively poor [181]. In short, inkjet printing is a scalable and promising technology for the future upgrading of large-scale PSCs and modules.

Table 1 presents the summary of different scalable deposition techniques used to prepare large-area perovskite thin films and their roll-to-roll capabilities, along with their current state-of-the-art device efficiencies for both modules (aperture area ≥10 cm^2^) and the single cells (aperture area ≤1 cm^2^).

All layer fabrication lines for PSCs may apply the combined multiple deposition methods integrated together. The most successful scalable solution technique to produce perovskites so far is blade coating, which only has demonstrated PCEs > 20%. Furthermore, the wastage of ink is substantially less than the other coating methods, particularly in a roll-to-roll deposition. Perovskite films fabricated with blade coating are typically uniform, pinhole-free, and display long-lived photoluminescence signifying it is a cost-effective deposition technique that can be tuned for a specific application. Though, significantly, variability in the quality coating might also be reported. Compared to other discussed methods, blade coating does not have arrangement for ink reservoir in its simplest form. So, the ink chemistry may change a little bit with the passage of some time. Similarly, spray coating and the slot-die coating methods are also scalable and roll-to-roll compatible. However, for the slot-die coating, the bottleneck is that a large quantity of ink used to fill the ink reservoir along with supply pipes is wasted. The same situation is with the spraying process; therefore, generally, both techniques are less utilized use to optimize the new ink chemistries. Consequently, the slot-die coating is less investigated, and the PSCs fabricated with slot die have a much lower efficiency than the device fabricated via blade coating. However, slot-die coating has the potential to demonstrate better results than blade coating when the ink is already fully developed. In the blade coating technique, thin-film morphology and device performance are mostly improved by the use of additives in the precursor solution. Therefore, judicial selection and addition of an optimized quantity of additives and optimizing the drying process may be promising techniques to improve the performance of devices fabricated by other scalable techniques. This process allows for a preferable crystallization pattern of the perovskite material with micrometer-large columnar grains.

## 6. Instability in Perovskite Solar Cells

Instability in PSCs is one of the biggest challenges in their commercial application. In the following paragraph, we have briefly reviewed prominent causes of instability and their solutions in PSCs.

### 6.1. Causes

Mostly, the metal electrodes are used for back contacts in PSCs, the corrosion on electrodes invasion badly affects the performance of the device. Similarly, the ions movement from both sides results in the degradation in perovskite and lowers the PCE [182,183]. Charged point defect migration and accumulation on perovskite and ETL or HTL interfaces badly affect fill factor and the PCE interfaces and grain boundaries are the places where degradation starts easily [184].

### 6.2. Solutions

Interfacial layers: Several approaches have been employed to improve device stability and performance. Inserting buffer layers between HTM and perovskite, replacement of instable HTM with some stable materials, and appropriate encapsulation can effectively improve stability [185]. Similarly, the interface modification [186,187,188] between the ETL and the perovskite absorber or using such materials which remain stable under UV radiation can improve stability [189]. Interfacial engineering is also an effective approach which has shown better stability. Such layers stop metal ion diffusion and helps to generate stable PSCs [185,190].

Use of additives: The interfaces and grain boundaries-sites are more vulnerable places where degradation may start easily, therefore, protecting them can slow the decomposition process. The use of additives such as quaternary ammonium halide [191], urea [112], thiophene, pyridine [116], and phenyl-C_61_-butyric acid methyl ester (PC_61_BM) [192] have shown the improvement in device stability [193,194]. The additive technique is most promising because it passivates defect states and stops instability causing channels.

Dimensionality engineering: Devices using low-dimensional perovskites in which some or all cations are replaced by large organic ligands displayed improvement in stability over the long-term operation against moisture and light soaking, as compared with 3D perovskite devices [180,195,196]. However, large ligands disrupt charge-transfer transport and sacrifice efficiency [197,198]. Alternatives such as conjugated cations [198] and shorter-chain cations [199], can inherently increase efficiency without sacrificing stability [200,201].

In the present scenario, the accurate estimation of standard stability of PSCs is difficult due to the different testing condition used by the PSC community [202]. The perovskite PV community needs to improve the stability reporting standards. Different light sources are used in indoor light stability tests [203]. To develop and follow the standard protocols, which address all the instability channels which may be enabled, accelerates stability and helps to accurately predict the lifetime of PSCs.

## 7. Conclusions and Outlook

Perovskite thin films have yielded exceptional photovoltaic performance and are considered compatible with inexpensive, scalable manufacturing. Despite significant improvements in small-area PSCs’ photovoltaic performance, the large-area fabrication of uniform and high-quality perovskite films remains a challenge. We have explored recent progress on the understanding of crystal growth mechanisms for the large-area fabrication of perovskite films and devices on this account. It has been demonstrated based on Fick’s first law that the solute particle diffusion process can control the crystal’s growth process. This prerequisite allows LaMer’s model to be applied in order to comprehend the perovskite growth mechanism. Therefore, it is commonly used to describe the morphological variation in perovskites, based on the understandings of nucleation and crystal growth. We have highlighted several physical and chemical techniques to control perovskite film morphology during the growth process. Antisolvent extraction is one of the most successful physical techniques for lab-scale devices. To meet the requirements of large area deposition, hot casting, vacuum quenching, and gas blowing are used individually or in combination as per the requirement of the deposition technique. Several chemical approaches have evolved for the spin-coating method, such as additives; compositional engineering has been found to still be useful in the scalable processes for improvement of film quality and optoelectronic properties of the large-area perovskite films. Noticeable progress has been made in the successful coating of perovskite absorber layers with the different scalable thin film coating technologies as described in this review. Comprehensive understandings of perovskite nucleation process as well as crystal growth theories, and the strategies applied during the deposition and growth processes to control the morphology of perovskite film have been summarized.

Understanding the crystallization process and underlying solution chemistry has enabled the deposition of better perovskite crystals and reproducible devices. Despite the great progress in the large-area fabrication of PSCs, the PCE of the large-area module is still well below the marketing threshold. The most successful scalable solution technique to produce perovskites so far is blade coating, which only has demonstrated PCEs > 20%. The deposition of back contacts of PSCs by vacuum-free processes is highly required to fully utilize the cheap processing advantages of PSCs. Although, the slot-die-coated PSCs with screen-printed silver electrodes produced only a little lower PCE than cells with evaporated metal (silver) electrodes (6.4% versus 7.9%), displaying the good potential of vacuum-free fabrication. Systematic analysis of solution chemistry and crystallization processes of the perovskite thin films with diverse coating methods would be necessary for further improvement in the photovoltaic performance of the large-area perovskite modules. For instance, molecular interactions of specific compositions in precursor solutions and their impacts on perovskite film crystallization are still under study. Knowing the precursors interaction and developed growth kinetic models would be helpful in designing a rational solution composition for film fabrication using various coating methods. This would also be essential to figure out how to adapt the advanced techniques established for both small area devices to the scalable devices. This review has sought to cover the detailed recent studies encompassing crystal growth theories, recent adopted growth technique, and scalable fabrication of PSCs. Hopefully, this review article can help understand the perovskite crystal growth mechanism and massive large-area fabrication of thin films for photovoltaic applications.

## Figures and Tables

**Table 1:** A summary of scalable solution processing technologies for perovskite solar cells.

Method	Material	Roll to Roll	Largest Coating Area (cm^2^)	PCE (%) $\mathrm{Single}\;\mathrm{Cell}\;\mathrm{Area}\leq 1\;({\mathrm{cm}}^{2})$	PCE (%) Module Area ≥ 10 (cm^2^)	Refs.
Blade coating	MAPbI_3_	yes	6 × 15	20.3	14.6	[78]
Slot die coating	MAPbI_3_	yes	12.5 × 13.5	~12	10	[152]
Spray coating	MAPbI_3_	yes	7.5 × 7.5	16.9	14.2	[164]
Ink jet printing	MAPbI_3_	yes	2.02	17.74	-	[175]
